# The nuclear exosome subunit HEN2 acts independently of the core exosome to assist transcription in Arabidopsis

**DOI:** 10.1093/plphys/kiae503

**Published:** 2024-09-25

**Authors:** Susheel Sagar Bhat, Mishaneh Asgari, Sarah Mermet, Priyanka Mishra, Peter Kindgren

**Affiliations:** Umeå Plant Science Centre, Department of Forest Genetics and Plant Physiology, Swedish University of Agricultural Sciences, 90187 Umea, Sweden; Umeå Plant Science Centre, Department of Forest Genetics and Plant Physiology, Swedish University of Agricultural Sciences, 90187 Umea, Sweden; Umeå Plant Science Centre, Department of Forest Genetics and Plant Physiology, Swedish University of Agricultural Sciences, 90187 Umea, Sweden; Umeå Plant Science Centre, Department of Forest Genetics and Plant Physiology, Swedish University of Agricultural Sciences, 90187 Umea, Sweden; Umeå Plant Science Centre, Department of Forest Genetics and Plant Physiology, Swedish University of Agricultural Sciences, 90187 Umea, Sweden

## Abstract

Regulation of gene expression is at the frontier of plant responses to various external stimuli including stress. RNA polymerase-based transcription and post-transcriptional degradation of RNA play vital roles in this regulation. Here, we show that HUA ENHANCER 2 (HEN2), a co-factor of the nuclear exosome complex, influences RNAPII transcription elongation in Arabidopsis (*Arabidopsis thaliana*) under cold conditions. Our results demonstrate that a *hen2* mutant is cold sensitive and undergoes substantial transcriptional changes compared to wild type when exposed to cold conditions. We found an accumulation of 5′ fragments from a subset of genes (including *C-repeat binding factors 1–3* [*CBF1–3*]) that do not carry over to their 3′ ends. In fact, *hen2* mutants have lower levels of full-length mRNA for a subset of genes. This distinct 5′-end accumulation and 3′-end depletion was not observed in other NEXT complex members or core exosome mutants, highlighting HEN2's distinctive role. We further used RNAPII-associated nascent RNA to confirm that the transcriptional phenotype is a result of lower active transcription specifically at the 3′ end of these genes in a *hen2* mutant. Taken together, our data point to the unique role of HEN2 in maintaining RNAPII transcription dynamics especially highlighted under cold stress.

## Teaser

HEN2, a nuclear exosome co-factor protein, plays a role in transcription independent of its role in degrading RNA in the plant cell.

## Introduction

Modulation of gene expression via transcriptional regulation is a mechanism essential for sustenance of life. Life forms change their transcriptional outputs in response to changing cellular needs (e.g. developmental cues) as well as external stimuli. Plants are especially adept at rapidly changing their transcriptional activity due to their sessile nature. A particularly challenging situation for the plant is when the temperature changes hastily. This has profound consequences on how the main transcription enzyme, RNA Polymerase II (RNAPII) can access the DNA around active genes and transcribe them (Kindgren et al. 2020). For a plant to adapt to colder temperatures, the chromatin and transcription must be modified. These processes are all part of the cold acclimation program that enables continued survival at low nonfreezing and freezing temperatures (Thomashow 1999). Cold acclimation is initiated within minutes of the plant sensing cold temperatures by the induction of a small set of transcription factors (TFs) (Thomashow 1999). Key among those TFs are the *C-REPEAT BINDING FACTOR 1–3* (*CBF1–3*) that are localized in tandem on chromosome 4 in the Arabidopsis (*Arabidopsis thaliana*) genome (Gilmour et al. 1998; Shinwari et al. 1998). The *CBF* region is the most induced genomic region in Arabidopsis in response to cold temperatures (Kindgren et al. 2018) and the genes are highly regulated, both to achieve transient peak expression but also their repression following the peak. The repression of *CBF1* and *CBF3* is partly regulated by the mechanisms of the long noncoding RNA, *SVALKA*, which is transcribed from the antisense strand between *CBF1* and *CBF3* (Kindgren et al. 2018; Gómez-Martínez et al. 2024; Zacharaki et al. 2023). The CBFs subsequently activate a larger set of cold regulated genes responsible for achieving cold hardiness (Thomashow 1999).

However, it is not enough to activate gene expression to adapt to cold temperatures. The produced RNA must also be processed via various processes (for example pre-mRNA splicing, miRNA and rRNA biogenesis) that are heavily affected by temperature (James et al. 2012; Hang et al. 2018; Ré et al. 2019). All these steps of RNA metabolism are regulated by RNA helicases, a ubiquitous family of enzymes that hydrolyze NTP to alter RNA structure and remodel ribonucleoprotein complexes (Li et al. 2023). Most plant genomes encode between 100 and 300 RNA helicases (Li et al. 2023). For example, Arabidopsis has at least 177 (Umate et al. 2010; Li et al. 2023). While we know many molecular mechanisms of RNA helicases in human and yeast cells, we have only begun mechanistic studies in plants (Li et al. 2023).

An important auxiliary role for RNA helicases is to activate the degradation of aberrant RNA transcripts in conjunction with the exosome, a 3′–5′ RNA exonuclease. Like metazoans and yeast, plants use a homolog of the RNA helicase, MRNA TRANSPORT4 (MTR4), as a co-factor of the nuclear exosome (de la Cruz et al. 1998; Lange et al. 2014). MTR4 is mainly involved in the degradation of rRNA biogenesis byproducts in plants, not in degradation of RNAPII transcripts, and thus has a more specialized role. In contrast to metazoans and yeast, a plant-specific RNA helicase, HUA ENHANCER 2 (HEN2), is responsible for degrading many mis-spliced, noncoding, and prematurely terminated RNAPII transcripts (Lange et al. 2014). HEN2 interacts with the core nuclear exosome, the cap-binding complex that binds to the cap on the 5′ end of newly synthesized RNA, and the nuclear exosome targeting (NEXT) complex (Lange et al. 2014). In metazoans, the NEXT complex guides RNA targeted for degradation to the exosome (Lubas et al. 2015). It consists of 3 subunits, the RNA binding protein, RBM7, and the zinc-knuckle protein ZCCHC8 together with MTR4 (Lubas et al. 2011). In Arabidopsis, HEN2 interacts with RBM7 and the 2 homologs of the human ZCCHC8, ZCCHC8A, and ZCCHC8B (Lange et al. 2014; Bajczyk et al. 2020). Although, the metazoan NEXT complex has been extensively studied (Lubas et al. 2011, 2015), little is known about the roles of the plant counterpart other than its role in miRNA processing (Bajczyk et al. 2020).

In this study, we investigated how the *hen2* mutants respond transcriptionally to cold temperatures. Previous work in Arabidopsis utilizing Transcript isoform sequencing (TIF-seq) in control and cold conditions revealed that the *hen2–2* mutant accumulates additional mRNA isoforms in cold, particularly short transcripts in the 5′ end of genes (Thomas et al. 2020). With RNA sequencing (RNA-seq), we detected a plethora of mis-regulated genes in *hen2–2* compared to wild type, particularly genes involved in stress response. We found that this mis-regulation was biologically important and resulted in an impaired cold acclimation process in *hen2–2* and other mutant alleles of *HEN2*. Deeper examination of steady state and nascent levels of mRNAs led us to discover a role for HEN2 as an RNAPII stimulant, important for the transcription complex to reach the end of a subset of cold induced genes. Importantly, this role was independent from the rest of the nuclear exosome and the NEXT complex, suggesting that HEN2 has additional critical roles other than those previously described.

## Results

### The hen2-2 mutant is cold sensitive

**Figure 1.**
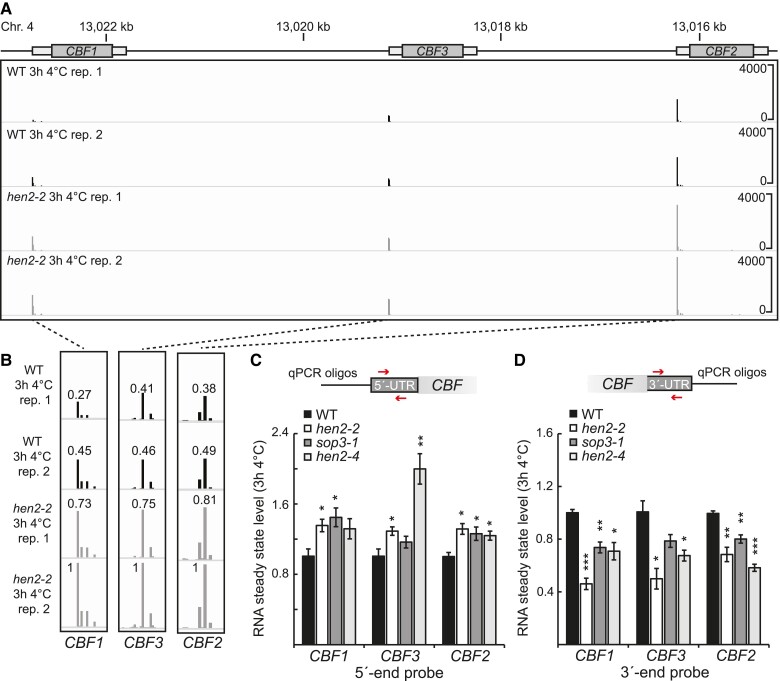
*Hen2* mutants accumulate 5′-end transcripts but have decreased levels of *CBF* mRNA in cold. **A)** Screenshots of TSS-seq data from 2 biological replicates of wild type Col-0 (WT) and *hen2-2*. **B)** Close up of the TSS of *CBF1-3* in wild type and *hen2-2* after 3 h at 4 °C. TSS signals have been normalized to the level of the second *hen2-2* replicate. **C and D)** The relative steady state level of the **C)** 5′ end, and **D)** 3′ end of *CBF1-3* measured with RT-qPCR in WT and *hen2* mutants at 22 °C compared to 3 h at 4 °C. Steady state levels were normalized to the WT levels at 22 °C. Arrows represent the placement of forward and reverse primers. The mean values are from 3 biological replicates. Error bars represent ± SEM. Statistical significance was calculated with Student's *t*-test (*P < 0.05, ***P < 0.001).

**Figure 2.**
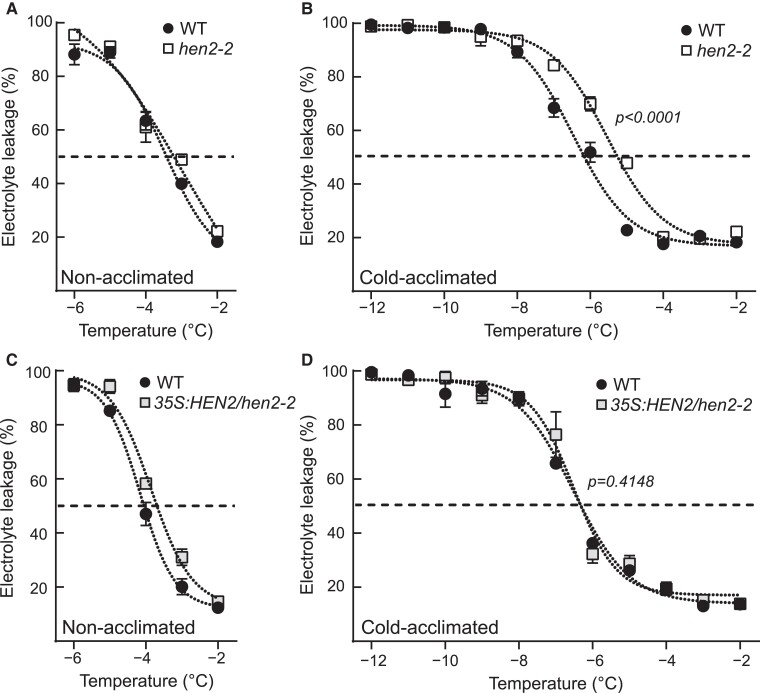
The *hen2-2* mutant is cold sensitive. **A and B)** Electrolyte leakage in wild type and *hen2-2* of **A)** non- and **B)** cold-acclimated (4 d of 4 °C, right panel) plants. Each data point represents the mean from at least 3 biological replicates (±SEM), and the data have been fitted to a sigmoidal response. Statistical difference between the curves was determined by an extra sum-of-squares F-test. **C and D)** Electrolyte leakage in wild type and complemented *hen2-2* of **C)** non- and **D)** cold-acclimated (4 d of 4 °C, right panel) plants. Each data point represents the mean from at least 3 biological replicates (±SEM), and the data have been fitted to a sigmoidal response. Statistical difference between the curves was determined by an extra sum-of-squares F-test.

The *hen2-2* mutant was recently shown to accumulate 5′-end transcripts of mRNAs, and this effect was exacerbated in cold temperatures (Lange et al. 2014; Thomas et al. 2020). We contemplated if this would have an overall impact on the cold acclimation process. Firstly, we investigated the *CBF* genes, known master regulators of the cold response in plants using available Transcription Start Site sequencing (TSS-seq) data after 3 h of 4 °C in wild type and *hen2-2*. Indeed, *CBF1-3* showed increased 5′-end signal compared to wild type (Fig. 1, A and B). These results corroborated TIF-seq data, both *CBF2* and *CBF3* showed accumulation of short proximal promoter RNAs (sppRNAs) (Thomas et al. 2020), and we could confirm it with reverse transcription quantitative PCR (RT-qPCR) for *hen2-2* and 2 additional mutant alleles for *HEN2* (Fig. 1C). Interestingly, when a 3′-end probe was amplified, we detected a decreased level of the *CBFs* after 3 h at 4 °C in the *hen2* mutants (Fig. 1D). This indicates that, although mutants of *HEN2* accumulate 5′-end transcripts from *CBF1-3*, the mutants do not have wild type levels of full-length mRNAs from *CBF1-3* after cold exposure. This further suggests that some initiated transcription events in *hen2* mutants do not reach the 3′ end of the *CBFs* resulting in an impaired cold response. To investigate if the mis-regulation of the *CBFs* had a biological consequence for the *hen2* mutants, we exposed non-acclimated and cold-acclimated *hen2* plants to freezing temperatures to measure the electrolyte leakage and compared it to wild type. In non-acclimated plants, there was no difference between wild type and *hen2* (Fig. 2A, Supplementary Fig. S1, A to D). However, in cold-acclimated plants, *hen2* mutants showed a decreased freezing tolerance (significantly more electrolyte leakage) compared to wild type (Fig. 2B, Supplementary Fig. S1, A to D), indicating an impaired cold acclimation process. To confirm that mutating *HEN2* is responsible for the cold acclimation defect, we complemented the *hen2-2* mutant with a 35S:*HEN2* construct and were able to restore the phenotype along with the restoration of WT like 3′-end levels of CBFs after cold stress (Fig. 2, C and D, Supplementary Fig. S1E). Taken together, these results suggest that *hen2* mutants are cold sensitive. Further, the accumulation of 5′-end isoforms of the *CBFs* in *hen2* mutants are accompanied by a decreased level of *CBF* full-length mRNA, indicating a complex mis-regulation of the *CBFs* in *hen2* mutants.

### HEN2 functions independent of other exosome subunits in regulating the CBF genes

**Figure 3.**
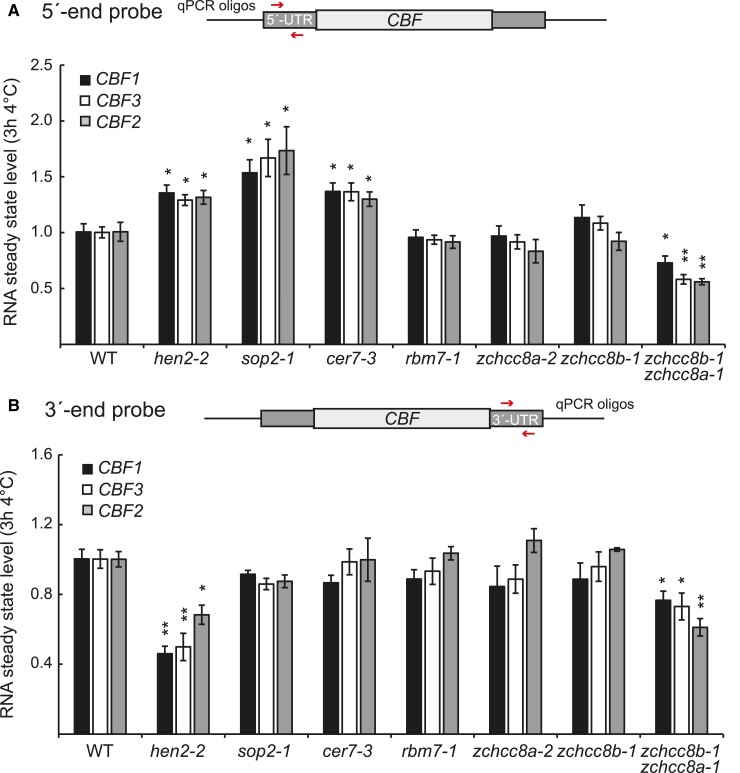
*hen2-2* functions independent of known interaction proteins to regulate the *CBFs*. **A and B)** The relative steady state level of the **A)** 5′ end, and **B)** 3′ end of *CBF1-3* measured with RT-qPCR in WT and mutants implicated in RNA degradation by the nuclear exosome at 3 h 4 °C. The locations of forward and reverse primers are represented by arrows. The mean values are from 3 biological replicates. Error bars represent ± SEM. Statistical significance from wild type was calculated with Student's *t*-test (*P < 0.05, **P < 0.01).

To investigate if the mis-regulation of the *CBF* genes was restricted to *hen2* mutants or shared with mutants of HEN2-interacting proteins, we isolated T-DNA lines from 3 NEXT complex subunits in Arabidopsis (*A. thaliana*) (*rbm7-1*, *zchcc8a-2*, *zchcc8b-1*, and *a zchcc8a-1/8b-1 double mutant*). We also included *sop2-1*, which has a point mutation in the core exosome subunit gene *RRP4* (Hématy et al. 2016), and *cer7-3*, a mutant in *RRP45A* (Hooker et al. 2007). We quantified 5′ and 3′ ends of the 3 *CBF* genes in all these mutants after cold treatment for 3 h at 4 °C. Interestingly, only *sop2-1* and *cer7-3* showed increased levels of 5′-end transcripts from *CBF1-3* (Fig. 3A). On the other end, neither *sop2-1* nor *cer7-3* showed any mis-regulation of full-length *CBF* mRNA (at the 3′ end, Fig. 3B). These results reinforced the role of the nuclear exosome in the degradation of prematurely terminated 5′-end transcripts from *CBF1-3*. None of the NEXT mutants had any mis-regulation of the *CBF* transcripts, except the *zchcc8* double mutant (Fig. 3, A and B). These results suggest that ZCHCC8A/B may be involved in regulating *CBF* mRNA levels, but not in the degradation of prematurely terminated transcription events. This role seems to be independent of the complete NEXT complex (which includes RBM7). Overall, our results suggest that the core nuclear exosome is responsible for the degradation of prematurely terminated 5′-end transcripts from *CBF1-3*, but it is not involved in regulating the level of full-length *CBF* mRNA. Thus, ZCHCC8A/B and HEN2 have evolved plant-specific functions independently from the core exosome and complete NEXT complex to regulate *CBF1-3* levels in response to cold.

### The genome wide transcriptional response in hen2-2 to cold temperature

**Figure 4.**
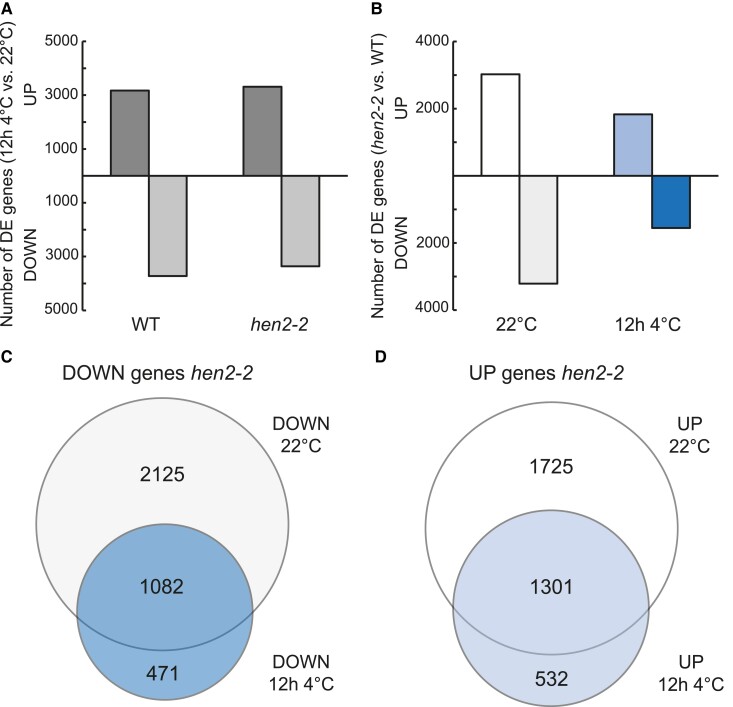
Stress-responsive genes are mis-regulated in the *hen2-2* mutant. **A)** Number of differentially expressed genes measured by RNA-seq in wild type (3172 UP and 3725 DOWN genes) and *hen2-2* (3311 UP and 3360 DOWN genes) in response to cold stress. Data obtained from 3 biological replicates. **B)** Number of differentially expressed genes in *hen2-2* compared to wild type at 22 °C (3026 UP and 3207 DOWN genes) and after 12 h at 4 °C. Data obtained from 3 biological replicates. **C)** Venn diagram comparing DOWN genes in *hen2-2* in 22 °C and after 12 h at 4 °C. **D)** Venn diagram comparing UP genes in *hen2-2* in 22 °C and after 12 h at 4 °C.

To get a full picture of how *hen2* mutants transcriptionally respond to cold temperatures, we performed an RNA-seq experiment using poly(A) enriched RNAs from *hen2-2* and wild type with 2 time points, a control point (10-d-old seedlings grown in long days and 22 °C) and after 12 h of cold treatment (4 °C). We chose 12 h at 4 °C for 2 reasons. Firstly, most cold regulated genes activated by the CBFs and other CBF-independent pathways responsible to establish the acclimatization process to cold temperatures are peaking around 12 to 24 h at 4 °C (Thomashow 1999). Secondly, the active transcription dynamics of switching seedlings to 4 °C is stabilized after 12 h (Kindgren et al. 2020). We first confirmed the downregulation of *HEN2* in the *hen2-2* mutant in our RNA-seq data (Supplementary Fig. S2). On the whole gene level and without bias to genotype-specific defects at the 5′ and 3′ ends, differentially expressed (DE) genes were roughly the same in response to cold, 3172 genes upregulated (UP) and 3725 genes downregulated (DOWN) in wild type and 3311 UP and 3360 DOWN in *hen2-2* (Fig. 4A, Supplementary Table S2). However, there was a large set of genes that were DE between the genotypes. At 22 °C, 3026 genes were UP and 3207 DOWN in *hen2-2* compared to wild type (Fig. 4B, Supplementary Table S3). After cold treatment, 1833 genes were UP and 1553 DOWN in *hen2-2* compared to wild type (Fig. 4B, Supplementary Table S3). Focusing on the DOWN genes, 2125 were unique to 22 °C and 471 unique to 12 h at 4 °C (Fig. 4C). For the UP genes, 1725 were unique to 22 °C and 532 unique to 12 h at 4 °C (Fig. 4D). The enriched GO terms for genes DE at 22 °C were focused on stress response (UP genes) and photosynthesis and stress response, including cold response (DOWN genes) (Supplementary Tables S4 and S5). At 12 h 4 °C, the GO terms were similar to 22 °C and enriched of genes in stress response (Supplementary Tables S6 and S7). DOWN genes included cold responsive genes. Examples of known stress induced genes (Fowler and Thomashow 2002; Ji et al. 2015; Yin et al. 2017) can be seen in Supplementary Fig. S3. Scatterplots for all DE genes showed a clear bias for higher expression in WT at both 22 °C and 12 h 4 °C (Supplementary Fig. S4, A and B). When specifically plotting *COR* genes targeted by the CBFs, we could detect a tendency in *hen2-2*, earlier seen in the *CBF* genes with higher RNA level in the 5′ end and lower level in the 3′ end (Supplementary Fig. S5A). Important to note is that many *COR* genes are mis-regulated at 22 °C in *hen2-2* (Supplementary Fig. S5A), thereby not specifically mis-regulated after cold exposure. A similar trend could be seen in all targets for *CBF1-3*, many targets have a lower RNA level at the 3′ end, resulting in lower full-length mRNA levels (Supplementary Fig. S5, B to D). Taken together, the mis-regulation of the *CBFs* and the overall lower mRNA level of differentially expressed genes give a compelling reason of why *hen2-2* is cold sensitive.

### A subset of genes is specifically downregulated in the 3′ end in hen2-2

**Figure 5.**
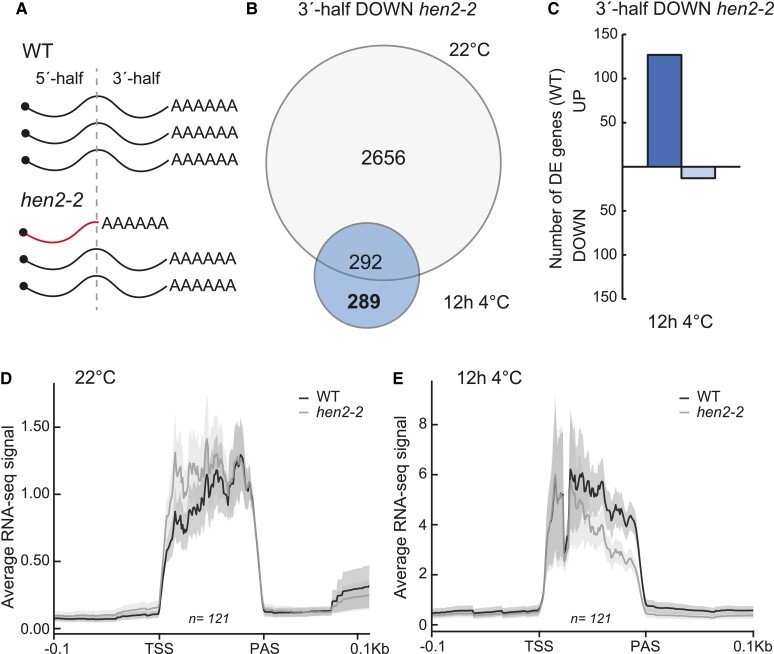
A subset of genes is mis-regulated in the 3′ end in *hen2-2*. **A)** Graphical representation of 5′-end accumulating transcripts and how they can mask the true DOWN genes in *hen2-2*. **B)** Venn diagram identifying genes that are specifically DOWN in the 3′ end in *hen2-2* compared to wild type after 12 h at 4 °C. Two hundred eighty-nine genes were identified. **C)** Number of DE genes in *hen2-2* that are UP or DOWN in WT after 12 h at 4 °C. Of the 289 DOWN genes in *hen2-2*, 127 were UP and 13 DOWN in WT. **D and E)** Metaplot of the average RNA-seq signal of the 3′-end DOWN *hen2-2*/UP in WT genes (*n* = 121). The plots show the RNA-seq signal at **D)** 22 °C and **E)** 12 h 4 °C.

**Figure 6.**
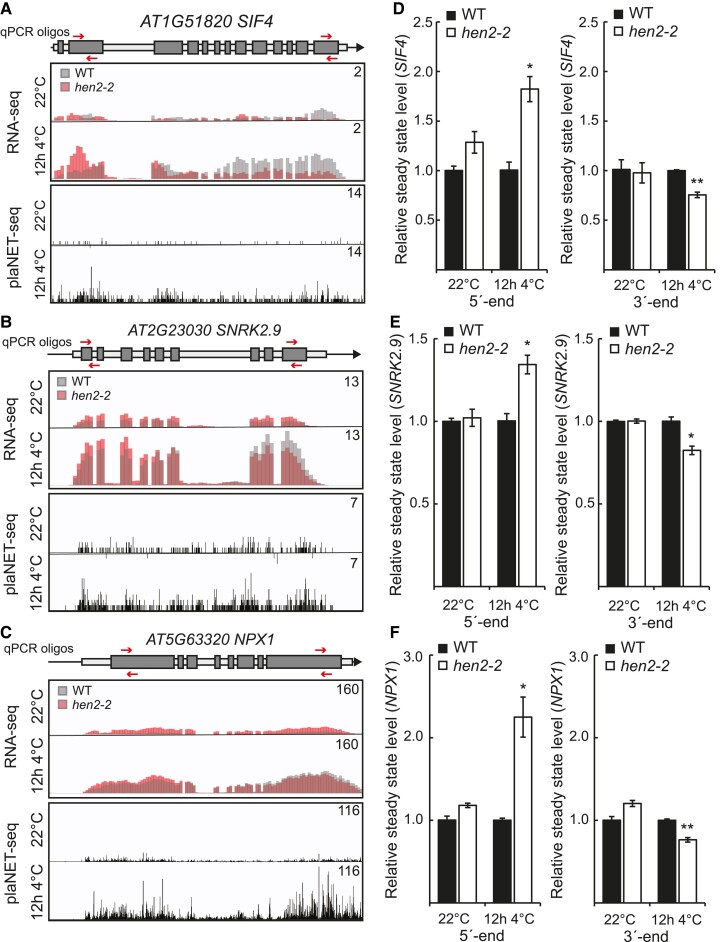
Candidate genes with 5′-end accumulation but decreased 3′-end mRNA level in *hen2-2*. **A to C)** Screenshot of overlayed RNA-seq data from wild type and *hen2-2* of **A)***SIF4*, **B)***SNRK2.9*, and **C)***NPX1* at 22 °C and 12 h at 4 °C. The position of the oligos used in **D to F)** is indicated on panels **A to D)** as arrows. Screenshots represent average reads obtained from 3 biological replicates. **D to F)** The relative steady state level of **D)***SIF4*, **E)***SNRK2.9*, and **F)***NPX1* measured with RT-qPCR in WT and *hen2-2* at the 5′ end (left panel) and 3′ end (right panel). Steady state levels were normalized to the WT levels at 22 °C and 12 h at 4 °C. The mean values are from 3 biological replicates. Error bars represent ± SEM. Statistical significance was calculated with Student's *t*-test (*P < 0.05, **P < 0.01).

Our results so far suggested that our initial RNA-seq analysis might not have been precise enough to detect all DE genes, specifically those that are activated in cold and accumulating 5′-end isoforms in *hen2-2*. Thus, it is possible that genes that are de facto DOWN in *hen2-2* are masked by increased 5′-end isoforms when looking at the whole gene level. Therefore, we re-run our RNA-seq analysis where we divided each gene in the middle, one 5′ end and one 3′ end (Fig. 5A). This simple division would make it possible to identify genes with a specific downregulation at the 3′ end, irrespective of any 5′-end *hen2* specific effects. We identified 289 genes that were specifically (i.e. not DE in control) DOWN in the 3′ end after 12 h at 4 °C in *hen2-2* (Fig. 5B, Supplementary Table S8). These genes tended to be long multi-exonic genes with multiple isoforms (Supplementary Fig. S6). Further, we inquired how these 289 genes would have responded in the presence of HEN2 (i.e. in WT). To this end, we matched them against how they responded to cold temperature in WT, measured by our RNA-seq data. One hundred twenty-seven genes were induced and only 13 genes were repressed (Fig. 5C, Supplementary Table S8), indicating that most genes in this subset were cold activated and this activation was hampered in *hen2-2* compared to wild type. Next, we plotted the average RNA-seq profiles of these genes at 22 °C and 12 h 4 °C (Fig. 5, D and E). There was a clear cold-specific 3′-end decrease at these genes in *hen2-2*, and the effect was gradually enhanced along the gene body (i.e. the difference was largest close to the PAS), indicating that *hen2-2* accumulates prematurely terminated isoforms throughout the transcription of the gene body after 12 h at 4 °C for these genes. We chose 3 genes from the list of 3′-end DOWN in *hen2-2* that had a visually convincing transcript isoform effect based on our RNA-seq data and showed a cold induced upregulation in RNA-seq and available nascent RNA levels (plaNET-seq) for a more thorough investigation (Fig. 6, A to C). Furthermore, we chose genes that had an increased level of 5′-end transcripts after 12 h at 4 °C in *hen2-2* compared to WT to better differentiate between WT and mutant. *STRESS INDUCED FACTOR 4* (*SIF4*) is a leucine-rich repeat-receptor-like kinase protein that has been studied in pathogen response (Zarattini et al. 2021). *SNF1-RELATED PROTEIN KINASE 2.9* (*SNRK2.9*) is involved in root growth and salt response (Kawa et al. 2020), and *NUCLEAR PROTEIN X1* (*NPX1*) responds to abscisic acid (Kim et al. 2009). None of the chosen genes had a previously known role in cold acclimation. The cold responsiveness of the genes was confirmed with RT-qPCR of independent samples (Supplementary Fig. S7) as well as the cold-specific transcript isoform effect of *hen2-2*, *sop3-1*, and *hen2-4* with 5′-end and 3′-end specific RT-qPCR amplicons (Fig. 6, D to F, Supplementary Fig. S8). Similar to the *CBFs*, the *sop2-1* and *cer7-3* mutants showed only increased 5′-end accumulation and not any 3′-end effect compared to WT (Supplementary Fig. S9). Our results suggest that a subset of cold induced genes in *hen2* mutants show a transcript isoform effect that may be incompatible with HEN2's described function as a post-transcriptional nuclear exosome co-factor. Instead, our data indicate that HEN2 has an additional co-transcriptional role, at least for the identified subset of genes, and this effect is independent of the exosome core.

### HEN2 assists active transcription at a subset of cold responsive genes

**Figure 7.**
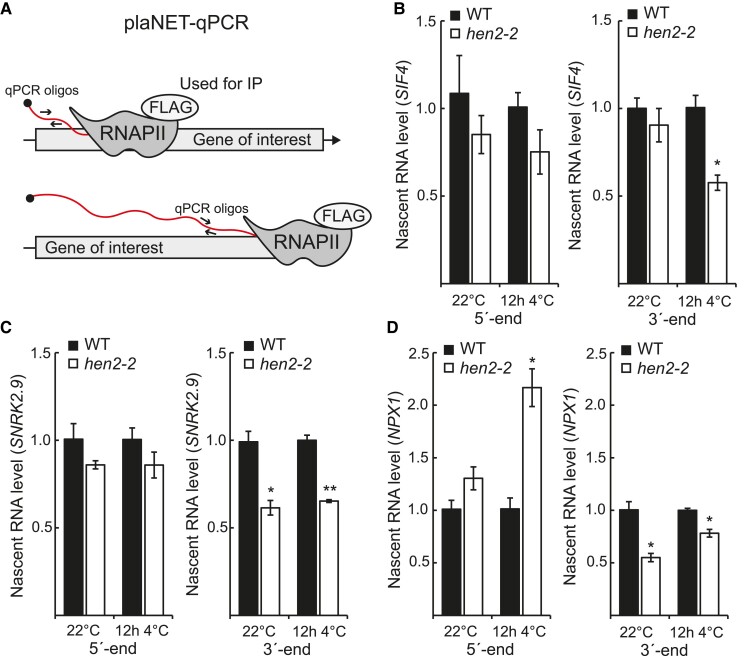
Active transcription reveals a RNAPII assisting role for HEN2. **A)** Graphical representation of the plaNET-qPCR technique. Oligos used for the 5′ end and 3′ end in **B to D)** are the same as in Fig. 6 and are represented by black arrows. Red line represents a transcribed mRNA. **B to D)** The nascent RNA level of **B)***SIF4*, **C)***SNRK2.9*, and **D)***NPX1* measured with plaNET-qPCR in WT and *hen2-2* at the 5′ end (left panel) and 3′ end (right panel). RNA levels were normalized to the WT levels at 22 °C and 12 h at 4 °C. The mean values are from 3 biological replicates. Error bars represent ± SEM. Statistical significance was calculated with Student's *t*-test (*P < 0.05, **P < 0.01).

To test our hypothesis that HEN2 has a co-transcriptional role, and to disentangle an initiation from an elongation role, we crossed the *hen2-2* mutant with a NRPB2-FLAG line covering a lethal *nrpb2-1* mutation (Onodera et al. 2008; Kindgren et al. 2018). *NRPB2* encodes the second largest subunit of RNA Polymerase II (RNAPII), the main RNAP in the plant cell. This mutant enables the isolation of actively transcribed RNA species still associated with RNAPII using an anti-FLAG antibody in the *hen2-2* background (Fig. 7A). Thus, we isolated RNA via immunoprecipitation using the FLAG-tag and RNA extraction from 3 independent biological samples at each time point for NRPB2-FLAG *nrpb2-1* (WT in Fig. 7) and NRPB2-FLAG *nrpb2-1 hen2-2* (*hen2-2* in Fig. 7). The RNA was used for cDNA synthesis using 5′-end and 3′-end gene-specific primers of our candidate genes and 2 reference genes followed by RT-qPCR. The primers were placed to avoid the main stall site around the +1 nucleosome and around the poly(A) signal to enable a measurement of active transcription. For *SIF4*, there was no difference at the 5′ end between *hen2-2* and wild type at 22 °C or 12 h at 4 °C (Fig. 7B). However, at the 3′ end, there was a significant decrease of transcribing RNAPII in *hen2-2* after 12 h at 4 °C. For *SNRK2.9* and *NPX1*, we detected no difference or higher RNA levels at the 5′ end but a lower RNAPII engagement level at both 22° and 12 h at 4 °C at the 3′ end (Fig. 7, C and D), indicating that the plant can compensate for its impaired RNAPII elongation at 22 °C since no effect is detected in the steady state levels. At 12 h 4 °C, however, the plant is no longer able to balance the transcriptional output due to stress. These results strongly support the hypothesis that HEN2 is required for RNAPII stimulation along the gene body of our candidate genes and not in the initiation of these genes. Moreover, this effect does not seem specific to 4 °C, at least not for *SNRK2.9* and *NPX1*, but rather a general effect on their transcription at multiple temperatures, albeit boosted in cold temperature due to the increased expression of the gene. Our results from the nascent RNA analysis combined with our other data strongly support a co-transcriptional role of HEN2.

## Discussion

**Figure 8.**
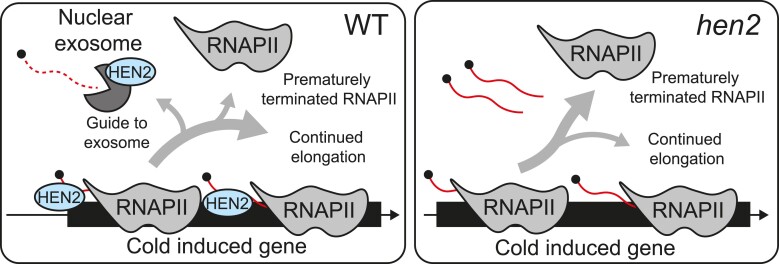
A working model of how HEN2 assists RNAPII. Working model for the positive regulation on RNAPII by HEN2. In wild type, HEN2 binds to the newly produced RNA (solid red) and assists RNAPII elongation. If RNAPII is terminated before it reaches the 3′ end of the gene, HEN2 guides the RNA for degradation by the nuclear exosome (dashed line). In *hen2-2*, RNAPII has a higher probability of premature termination and does not produce enough full-length mRNA for *hen2-2* to achieve proper cold acclimation.

Our results can be summarized in a working model where HEN2 acts independently from the nuclear exosome to regulate RNAPII elongation at a subset of genes that are induced by cold temperature (Fig. 8). We propose that, at these genes, in addition to acting as an RNA targeting guide for the nuclear exosome, HEN2 also influences RNAPII elongation. This assisting role has important consequences for the overall fitness of plants responding to cold stress. In the absence of HEN2, the probability of a prematurely terminated RNAPII increases at a subset of genes, resulting in a decreased amount of full-length mRNA. To have a promoting effect on RNAPII, HEN2 needs to bind or be close to the nascent RNA that exits the polymerase. It is unlikely that HEN2 has a strong direct interaction with RNAPII, thorough reciprocal co-immunoprecipitation experiments showed no contact (Lange et al. 2014). Nevertheless, HEN2 interacts with the cap-binding complex (CBC) that binds to the cap on the 5′ end of newly synthesized RNA exiting RNAPII (Lange et al. 2014). This interaction provides a direct coupling of HEN2 to nascent RNA. In further support of our model, HEN2 is localized to speckles in the nucleoplasm, in addition to a more diffuse localization in the nucleoplasm (Hématy et al. 2016). A core subunit of the nuclear exosome did not show any localization to speckles in the nucleoplasm (Hématy et al. 2016), again hinting of an independent role for HEN2. Our results showed that 5′ ends of transcripts accumulate only in *hen2* and nuclear exosome mutants. The accumulation can be explained by the role of the exosome in 3′ to 5′ degradation. However, we observed that only *hen2* mutants showed a decrease in transcript levels at their 3′ ends. This suggests that HEN2 may have an independent role without engaging the exosome core. A putative interaction between HEN2 and the nascent RNA would be an elegant way for the plant to guide any prematurely terminated mRNAs for exosome degradation and bypassing the NEXT complex and needs further investigation.

In our study, we have used cold temperature as a differentiator to induce changes to the transcriptional output. Plants have an amazing ability to compensate irregularities in mRNA levels when grown in control conditions. Therefore, stress cues induce effects that are normally challenging to detect in a normal growing environment. At first glance, the higher RNA level of the *CBF* genes after cold stress should render the *hen2-2* mutant more resistant to cold. However, we observed *hen2-2* to be more sensitive to cold. Further investigation clarified that the 5′-end accumulation of the *CBF1-3* did not carry over to their 3′ ends, explaining our results. The *hen2-2* mutant has earlier been shown to accumulate 5′-end isoforms of *CBF1* and read-though transcripts from the noncoding RNA, *SVALKA*, in response to the RNAPII collisions. *SVALKA* is transcribed from the antisense strand of *CBF1* (Kindgren et al. 2018). However, the 5′-end isoform accumulation in *hen2-2* predates the repressive RNAPII collision mechanism, suggesting that accumulation of prematurely terminated *CBF1* mRNA is occurring before *CBF1* is reaching peak expression (Kindgren et al. 2018; Thomas et al. 2020). It is possible that the accumulation of read-through *SVALKA* transcription in *hen2* further exuberates the cold-sensitive phenotype in *hen2* since it is complementary to the mRNA of *CBF1* and able to downregulate *CBF1* (Zacharaki et al. 2023). However, we do not see that *CBF1* is DE in our RNA-seq data at 12 h 4 °C in *hen2-2*. In fact, 5′-end isoform accumulation is not restricted to the early cold response (3 to 4 h of 4 °C), our RNA-seq data clearly show that this phenomenon is present after 12 h at 4 °C as well. Through our approach we could also identify and confirm 3 more cold responsive genes later in the cold response (*SIF4*, *SNRK2.9*, and *NPX1*) whose transcription is affected in *hen2* mutants.

How RNA transcripts targeted for degradation by the exosome are guided there is largely unknown, particularly in plants. In human cells, the NEXT complex subunit RBM7 have been suggested to fulfill a similar role to the proposed role of HEN2 in this study, although human RBM7 has not so far been found to stimulate RNAPII (Lubas et al. 2015). According to the model, RBM7 interacts with CBC to bind nascent RNAPII transcripts and, together with MTR4, direct them to the exosome if prematurely terminated (Lubas et al. 2015; Gerlach et al. 2022). In our study, the cold stressed *rbm7-1* mutant did not show any accumulation of 5′-end isoforms from *CBF1-3* and no decrease in full-length mRNA. This indicates that there could very well be a divergence and specialization in function between the human and plant proteins. RBM7 in Arabidopsis has been found to be involved in miRNA processing, together with HEN2, but not so far in general transcription (Bajczyk et al. 2020). The plant-specific evolution for these proteins is further corroborated with our results from the other NEXT subunit, ZCHCC8A/B. The *zchcc8a/b* double mutant showed a mis-regulation of the *CBFs*, indicating a role independent of RBM7. The putative link between HEN2 and ZCHCC8 to control *CBF* mRNA levels is an intriguing avenue for future research. Additionally, several pieces of data suggest that the 5′-end isoforms that accumulate in *hen2* mutants use an alternative to NEXT to guide these RNAs to the exosome. The Poly(A) exosome targeting (PAXT) complex is so far understudied in plants but promotes the degradation of polyadenylated transcripts (Lange and Gagliardi 2022). Both our RNA-seq and available TSS-seq and TIF-seq data sets are enriched for polyadenylated RNAs, suggesting that the 5′-end accumulated transcripts indeed contain a poly(A)-tail. Our data put HEN2 as a key player in the guiding of RNAs for degradation, but much remains unknown of the molecular mechanisms and interaction partners that assist this pathway.

An important question that emerges from our model is how HEN2 assists RNAPII transcription. There is increasing evidence that RNA helicases are important transcription regulators (Fuller-Pace 2006; Fuller-Pace and Nicol 2012; Sergeeva and Zatsepin 2021), but few examples are so far known in plants. Our results promote a role for HEN2 during active elongation of RNAPII, not initiation where several other RNA helicases have an implicated role in human cells (Giraud et al. 2018). Thus, the simplest example would be a situation where elongating RNAPII is stalled because of RNA structures formed by the nascent RNA. This would in turn require an RNA helicase to unwind the RNA so that transcription can proceed (Russon et al. 2021). We found that *hen2* mutants mis-regulate predominantly long genes with multiple exons and splicing contributes to major intragenic stall sites for RNAPII (Kindgren et al. 2020). Another possible link between RNAPII stalling, RNA helicase activity, and nascent RNA structure comes from human cells. RNA Guanine Quadruplexes (RNA G4) and DNA:RNA hybrids (R-loops) stall the RNAPII (Oh et al. 2021) and require RNA helicases for elongation to continue (Russon et al. 2021). Both RNA G4 and R-loops are common features in the Arabidopsis genome (Xu et al. 2017; Yang et al. 2020a, 2020b). Intriguingly, R-loops are most prevalent in 5′ ends of genes in Arabidopsis (Xu et al. 2017), corresponding with the accumulation of 5′ isoforms in *hen2-2* (Thomas et al. 2020). One of few studies from plants has characterized an RNA helicase responsible for unwinding R-loops in the chloroplast genome (Yang et al. 2020a, 2020b). An interesting path for future research is therefore to investigate any accumulation of RNA:RNA or RNA:DNA features in *hen2* mutants.

In conclusion, we propose a role for HEN2 that is independent of its role in the nuclear exosome. By stimulating RNAPII transcription, HEN2 enables stress-responsive genes to produce the proper amount of full-length mRNA to acclimatize to new conditions.

## Methods and material

### Plant material, growth conditions, and treatments

Arabidopsis (*A. thaliana*) wild type (Col-0) was used in this study. Additional mutants used have been described elsewhere, *hen2-2* (GABI_747H07) (Lange et al. 2014), *sop2-1* and *sop3-1* (EMS point mutations) (Hématy et al. 2016), *cer7-3* (GABI_089C02) (Hooker et al. 2007), *rbm7-1* (SALK_005077) (Bajczyk et al. 2020), *zchcc8a-2* (GABI_598C06) (Bajczyk et al. 2020), *zchcc8b-1* (GABI_667A12), and *zchcc8a-1* (SAIL_1230_H09) *zchcc8b-1* double mutant (Bajczyk et al. 2020). Seeds were sterilized using 70% ethanol and 0.05% Triton-X, air dried, and sown on ½ MS media pH 5.7, 1% sucrose, and 1% agar. Plates were kept in the dark at 4 °C for 48 h for stratification and then moved to a growth chamber with ∼100 µEm^−2^ s^−1^ light on long day (16 h light/8 h dark, 22 °C day/18 °C night) conditions. Ten-day-old seedlings were collected either directly or post-cold treatment (4 °C with ∼20 to 25 µEm^−2^ s^−1^ light). Three independent biological replicates were collected, flash frozen in liquid nitrogen, and stored at −80 °C till further use. For weighing of plants at control and 10 °C, plants were grown in short day conditions with ∼100 µEm^−2^ s^−1^ light on long day (8 h light/16 h dark, 22 °C day/18 °C night) for 3 wk and then kept at control or transferred to 10 °C with similar light conditions. Plants were weighed after 14 d after transfer.

### Cloning and complementation of hen2-2

cDNA of *HEN2* (AT2G06990) was cloned in a pDONR vector using gene-specific primers (Supplementary Table S1) based on Gateway technology (Life technologies). After initial cloning, coding sequence was cloned into expression vector allowing in planta expression under the 35S promoter (pGWB606, GFP Nter) (Nakagawa et al. 2007). The expression vector was transformed in the *Agrobacterium tumefaciens* strain GV3101 and used to reach stable transformation of *HEN2* in the *hen2-2* mutant (GABI_747H07). T2 plants with rescued phenotype were used to perform freezing test and RT-qPCR, respectively.

### RNA isolation, cDNA preparation, and RT-qPCR

RNA was isolated from ground tissue using EZNA Plant RNa kit (Omega Bio-tek) according to manufacturer's instructions. RNA obtained was treated with TURBO DNAse (Thermo Fischer) according to the standard protocol. For cDNA synthesis, iScript cDNA Synthesis Kit (Bio-Rad) was used. cDNA was synthesized using 500 ng of RNA as per manufacturer's protocol. qPCR was performed using iTaq Universal SYBR Green Supermix (Bio-Rad) and Bio-Rad CFX96/CFX384 Touch Real-Time PCR Detection Systems. For differential expression analysis, ΔCt was calculated (Ct of target − Ct of reference) and ΔΔCt was calculated (ΔCt mutant − ΔCt WT) followed by fold change (2^−ΔΔCt^). *ACTIN2* (*ACT2*, AT3G18780) and *UBIQUITIN 10* (*UBQ10*, AT4G05320) were used as reference genes. Statistical differences were calculated using Student's *t*-test. All oligos used in this study can be found in Supplementary Table S1.

### RNA sequencing and analysis

High quality RNA (RIN > 8) was sent to Novogene Europe for library preparation and sequencing. Strand specific libraries were generated at Novogene after polyA enrichment. Sequencing was performed on Illumina's NovaSeq 6000 platform, and 6 GB of raw data was obtained per sample. For data analysis, the guidelines previously established at UPSC were followed (Delhomme et al. 2023). Briefly, data were preprocessed and quality checked FastQC v0.11.9 (quality control of the raw data) and SortMeRNA v4.3.4 (filter and remove rRNA contamination) (Andrews 2010; Kopylova et al. 2012). Thereafter, Trimmomatic (Bolger et al. 2014) was used to trim the adapter sequences and FastQC was performed again to ensure data integrity. Salmon v1.6.0 (Patro et al. 2017) was used to determine the read counts with AtRTD2 (Zhang et al. 2017) as a reference. The salmon files obtained were used for the differential expression analysis using the 3D RNA-seq App (run locally, but also available online at https://3drnaseq.hutton.ac.uk/app_direct/3DRNAseq/) (Guo et al. 2021). GO-term analysis was done using PANTHER overrepresentation test (Fisher's exact test with FDR correction) (Mi et al. 2019). Publicly available TSS-seq data were obtained and analyzed according to previous reports (Kindgren et al. 2018; Thomas et al. 2020). Metaplots were prepared using cloud-based Galaxy interface (usegalaxy.org) (Afgan et al. 2016). Briefly, genomic loci for 121 genes of interest (6 were removed from the initial list because of adjacent genes influencing the signal) were filtered from Araport 11. These attributes, along with their expression data from our RNA-seq experiment, were used to generate a data matrix (computeMatrix) with genomic regions scaled to 100 bp with bin size of 1 bp. The statistics displayed correspond to the means calculated across each bin. Characterization of genes down at the 3′ end in *hen2-2* was done using ShinyGO 0.77 using *A. thaliana* genome as the background (Ge et al. 2020). To make scatterplots, read counts of target genes (genes DE in *hen2-2* vs WT at 22 °C and after 12 h at 4 °C) were calculated using FeatureCounts (https://doi.org/10.1093/bioinformatics/btt656) on usegalaxy.org. Read counts thus obtained were averaged for 3 biological replicates and used for making scatterplots using ggplot2 (Wickham 2016).

### Measurement of nascent RNA levels

The *hen2-2* mutant was crossed with a *nrpb2-1* mutant complemented with a *NRPB2:NRPB2*-FLAG construct as described (Onodera et al. 2008). Confirmed triple mutants were used to isolate RNAPII complexes. Firstly, nuclei were isolated and RNAPII complexes immunoprecipitated with the FLAG-tag according to (Kindgren et al. 2018) with a few modifications. To disrupt the RNAPII complexes, QIAzol was added directly to the beads, and RNA was isolated using the miRNeasy kit from Qiagen. RNA concentration was measured with Qubit, and approximately 100 ng was used for cDNA synthesis with gene-specific primers and Superscript IV (Invitrogen) according to manufacturer's instructions. Oligos used can be found in Supplementary Table S1.

### Freezing test

Electrolyte leakage measurements were carried out according to Kindgren et al. (2015). Five-week-old plants grown in short day conditions were used for the experiments. For the cold acclimation experiments, WT and *hen2-2* plants were transferred to a cold chamber set at 4 °C for 4 d. The freezing bath (FP51, Julabo) was set to −2 °C when the experiment started. After 60 min, icing was induced manually in each tube with a metallic stick. Temperature decrease occurred at the rate of −1 °C per 30 min, and samples were taken out at designated temperature point(s). When all samples had been collected, 1.3 ml of distilled water was added to each tube and placed on a shaker overnight at 4 °C and conductivity was measured using a conductivity cell (CDM210, Radiometer) on the next day. All tubes were then subjected to flash freeze and left on a shaker overnight at room temperature and measured for conductivity again. Data were fitted into a sigmoidal dose–response curve using GraphPad Prism software. Statistical significance between LogEC50 was calculated with an extra sum-of-squares F-test included in GraphPad Prism.

### Accession numbers

Sequence data from this article can be found in the ENA data libraries under accession number PRJEB67912. Data pertaining to the genes of interest in this study are provided in Supplementary Table S1.

## Supplementary Material

kiae503_Supplementary_Data

## Data Availability

RNA Seq data produced during this study are submitted and available online at ENA under accession number PRJEB67912.

## Supplementary data

The following materials are available in the online version of this article.

**Supplementary Figure S1.** Electrolyte leakage assay of 2 additional *HEN2* alleles (*sop3-1* and *hen2-4*) and RT-qPCR for 3′ ends of *CBF1-3* in complemented *hen2-2* line.

**Supplementary Figure S2.** Screenshot of browser view on the *HEN2* gene in WT and *hen2-2*.

**Supplementary Figure S3.** Screenshots of browser view for known stress induced genes.

**Supplementary Figure S4.** Scatterplots of genes DE in *hen2-2* at 22 °C and after 12 h at 4 °C.

**Supplementary Figure S5.** Metaplots of average RNA-seq signals of *COR* genes and CBF1-3 targets.

**Supplementary Figure S6.** Characteristics of genes down only at the 3′end in *hen2-2*.

**Supplementary Figure S7.** RT-qPCR for novel cold responsive genes.

**Supplementary Figure S8.** RT-qPCR for cold-specific transcript isoform effect with 5′ and 3′ specific primers on genes identified in Supplementary Fig. S7 in *hen2* mutant alleles.

**Supplementary Figure S9.** RT-qPCR showing the lack cold-specific transcript isoform effect with 5′ and 3′ specific primers on genes identified in Supplementary Fig. S7 in exosome mutant alleles.

**Supplementary Table S1.** Oligos used in this study.

**Supplementary Table S2.** Differentially expressed genes in wild type and hen2-2 after 12 h at 4 °C.

**Supplementary Table S3.** Differentially expressed genes between wild type and hen2-2 at 22 °C and after 12 h at 4 °C.

**Supplementary Table S4.** Enriched GO terms of DE (UP) genes in *hen2-2* at 22 °C.

**Supplementary Table S5.** Enriched GO terms of DE (DOWN) genes in *hen2-2* at 22 °C.

**Supplementary Table S6.** Enriched GO terms of DE (DOWN) genes in *hen2-2* at 12 h 4 °C.

**Supplementary Table S7.** Enriched GO terms of DE (UP) genes in hen2-2 at 12 h 4 °C.

**Supplementary Table S8.** Identification of candidate genes used for plots in Fig. 5, D and E.

## Dive Curated Terms

The following phenotypic, genotypic, and functional terms are of significance to the work described in this paper:

NRPB2 Gramene: AT4G21710
NRPB2 Araport: AT4G21710

RRP45A Gramene: AT3G12990
RRP45A Araport: AT3G12990

UBQ10 Gramene: AT4G05320
UBQ10 Araport: AT4G05320

RBM7 Gramene: At4g10110
RBM7 Araport: At4g10110

CBF1 Gramene: AT4G25490
CBF1 Araport: AT4G25490

ACT2 Gramene: AT3G18780
ACT2 Araport: AT3G18780

CBF2 Gramene: AT4G25470
CBF2 Araport: AT4G25470

HEN2 Gramene: AT2G06990
HEN2 Araport: AT2G06990

RRP4 Gramene: AT1G03360
RRP4 Araport: AT1G03360

ZCCHC8A Gramene: AT5G38600
ZCCHC8A Araport: AT5G38600

ZCCHC8B Gramene: At1g67210
ZCCHC8B Araport: At1g67210
