# Supplementary material for: The nuclear exosome subunit HEN2 acts independently of the core exosome to assist transcription in Arabidopsis
Source: Plant Physiol. 2024 Sep 25;196(4):2625–37. doi: 10.1093/plphys/kiae503 (PMC11638103; doi:10.1093/plphys/kiae503)
Supplement: kiae503_Supplementary_Data [file kiae503_supplementary_data.zip › PP2024RA00978DR1_Supplementary_Figures_1_9.pdf]

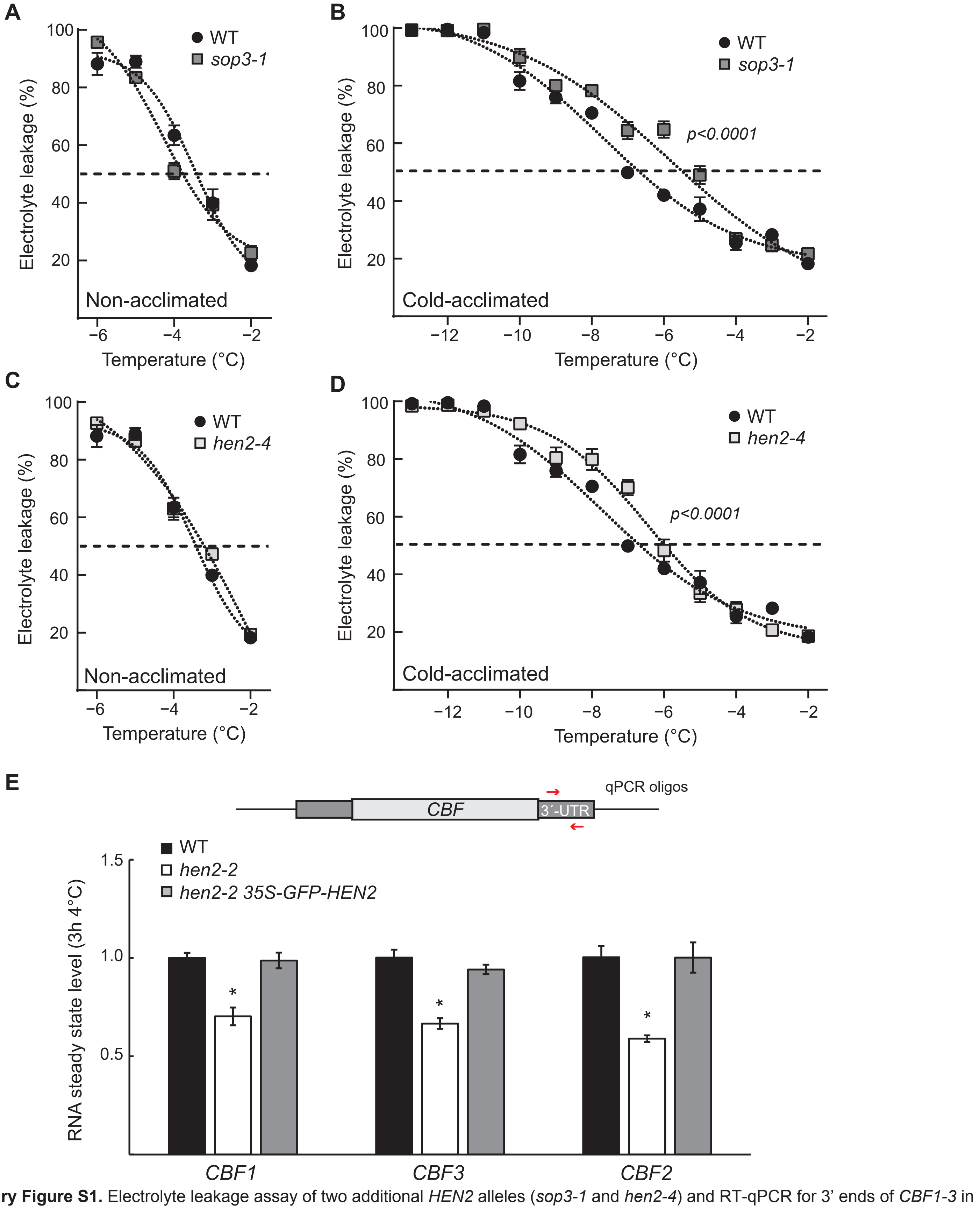

Supplementary Figure S1. Electrolyte leakage assay of two additional *HEN2* alleles (*sop3-1* and *hen2-4*) and RT-qPCR for 3' ends of *CBF1-3* in a complemented *hen2-2* line. A-B) Electrolyte leakage in wild-type and *sop3-1* of A) non- and B) cold-acclimated (4 days of 4°C, right panel) plants. Each data point represents the mean from at least 3 biological replicates (±SEM) and the data has been fitted to a sigmoidal response. Statistical difference between the curves was determined by an extra sum-of-squares F-test. (C-D) Electrolyte leakage in wild-type and *hen2-4* of C) non- and D) cold-acclimated (4 days of 4°C, right panel) plants. Each data point represents the mean from at least 3 biological replicates (±SEM) and the data has been fitted to a sigmoidal response. Statistical difference between the curves was determined by an extra sum-of-squares F-test. (E) The relative steady state level of the 3'-end of *CBF1-3* measured with RT-qPCR in wild type and complemented *hen2-2* at 3h 4°C. Primer locations are shown by red arrows. The mean values are from three biological replicates. Error bars represent ± SEM. Statistical significance from wild type was calculated with Student's t-test (*p<0.05).

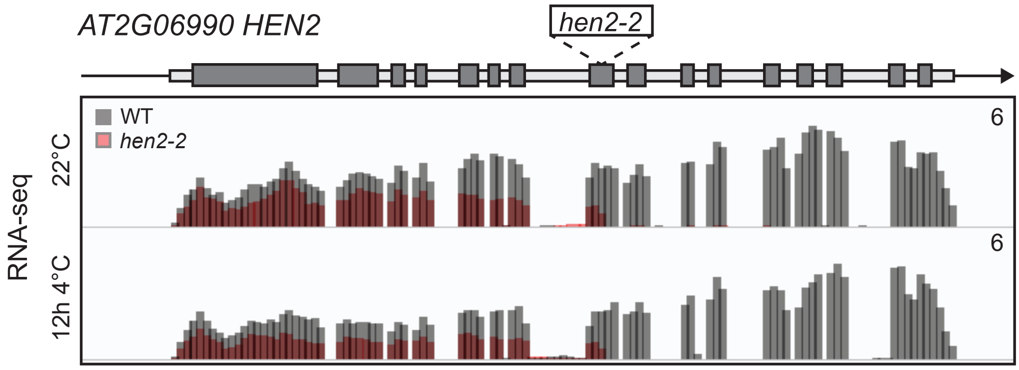

Supplementary Figure S2. Screenshot of browser view on the *HEN2* gene in WT and *hen2-2*. Screenshot of overlayed RNA-seq data from three biological replicates of wild type and *hen2-2* of *HEN2* (AT2G06990) at 22°C and 12h at 4°C. The position of the T-DNA insertion in hen2-2 is indicated.

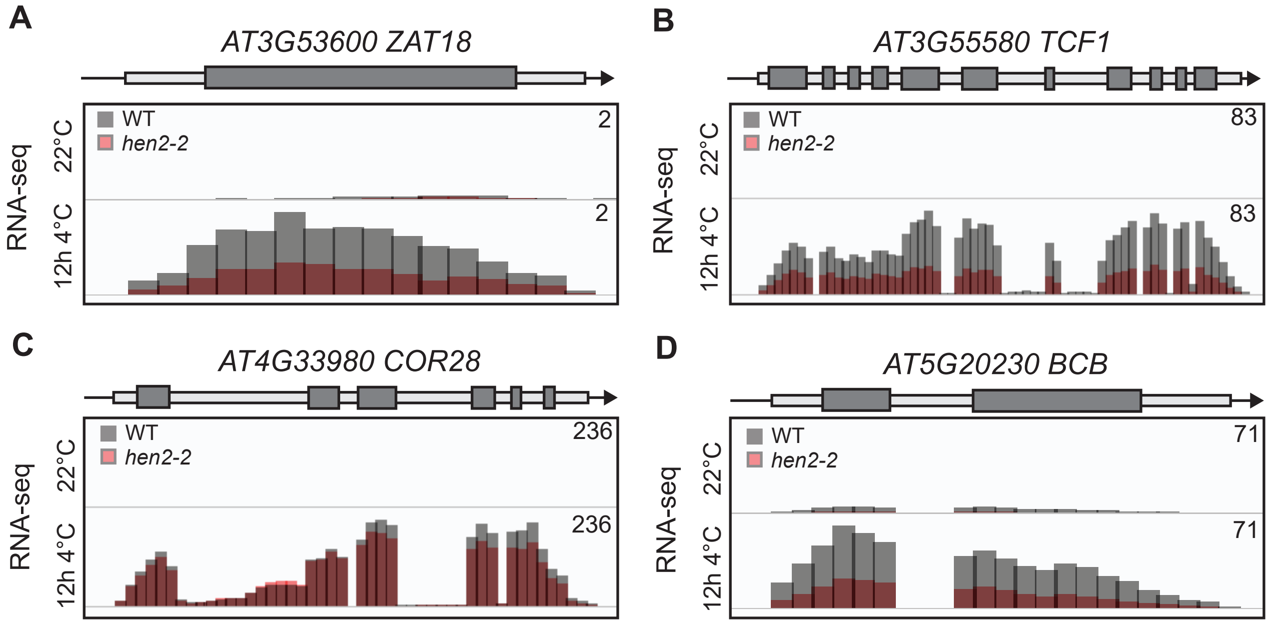

Supplementary Figure S3. Screenshots of browser view for known stress induced genes
A-D) Screenshot of overlayed RNA-seq data (obtained from three biological replicates) at 22°C and 12h at 4°C from wild type and *hen2-2* of A) *ZAT18* (AT3G53600), B) *TCF1* (AT3G55580), C) *COR28* (AT4G33980), D) *BCB* (AT5G20230).

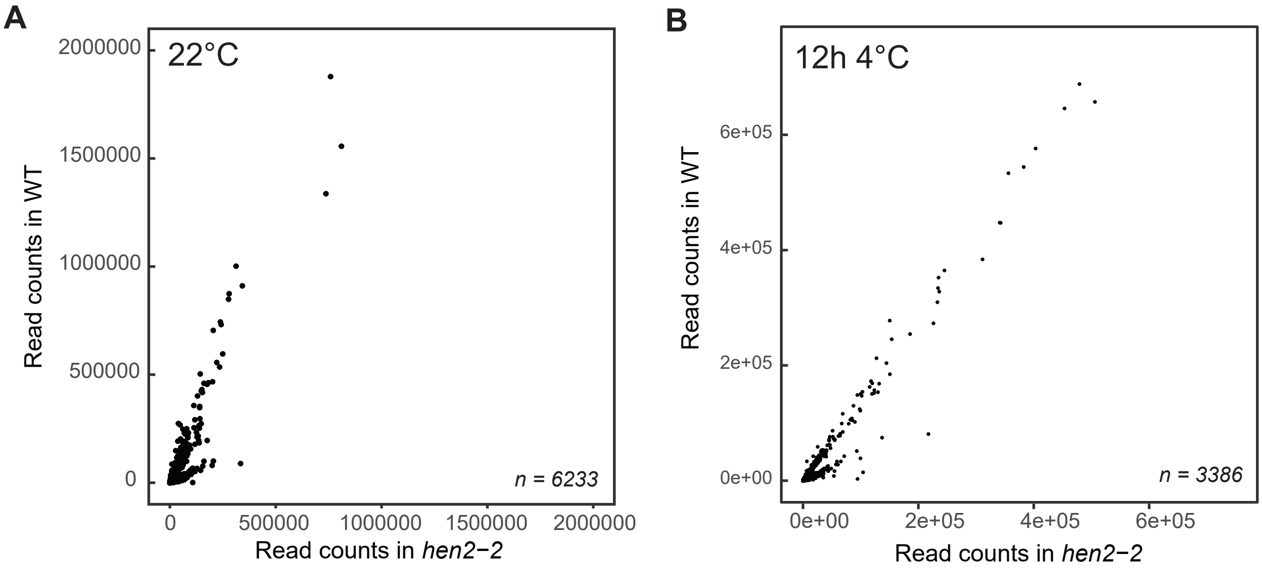

Supplementary Figure S4. Scatterplots of genes DE in *hen2-2* at 22°C and after 12h at 4°C.
A-B) Scatterplots of genes DE in *hen2-2* at 22°C A) and after 12h at 4°C B). x-axis: read counts in *hen2-2*; y-axis: read counts in WT. Read counts average of three biological replicates were used.

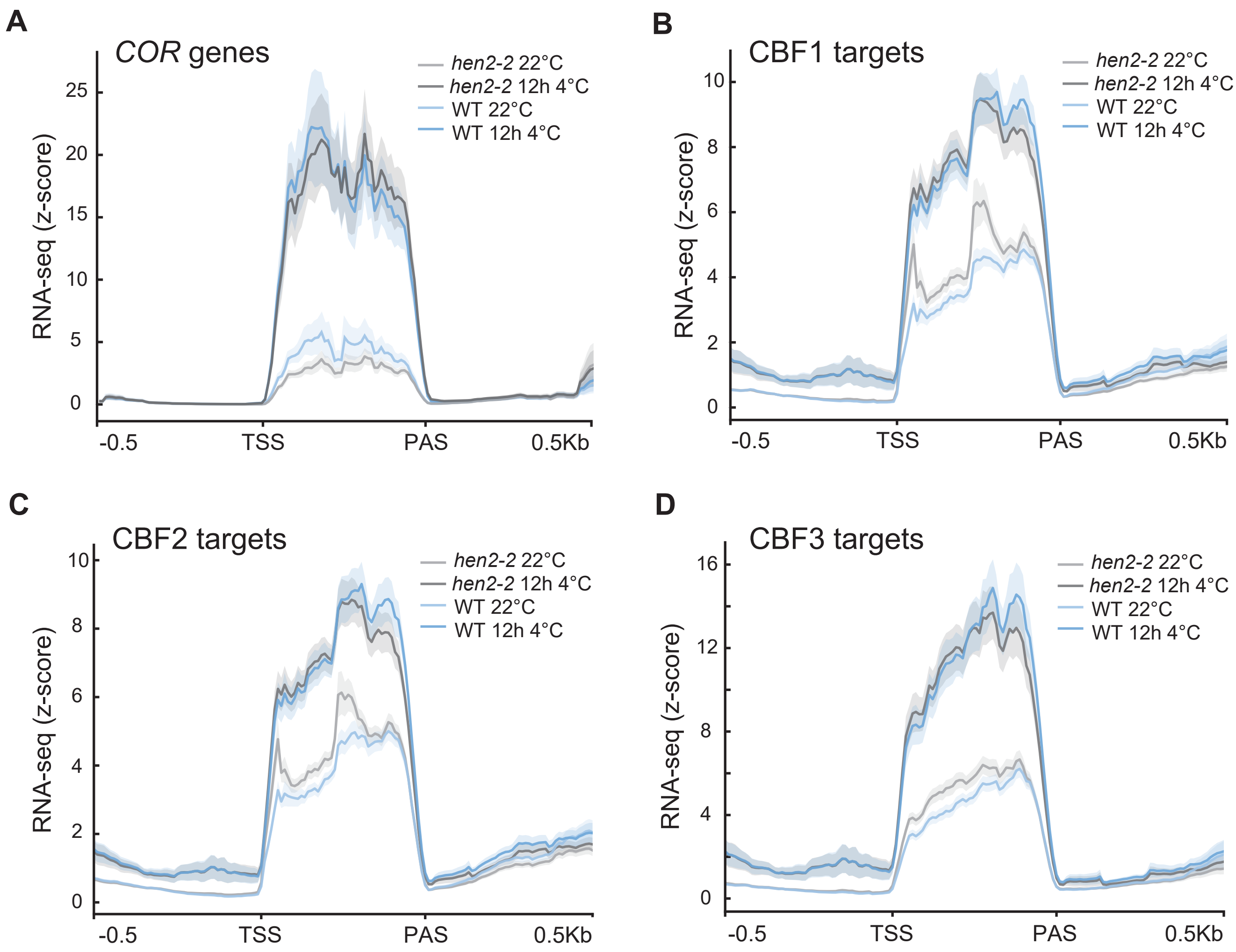

Supplementary Figure S5. Metaplots of average RNA-Seq signals of *COR* genes and CBF1-3 targets.
A-D) Metaplots of the average RNA-seq signal of A) *COR* genes, B) CBF1 targets, C) CBF2 targets, and D) CBF3 targets in wild type and *hen2-2* at 22°C and after 12h at 4°C.

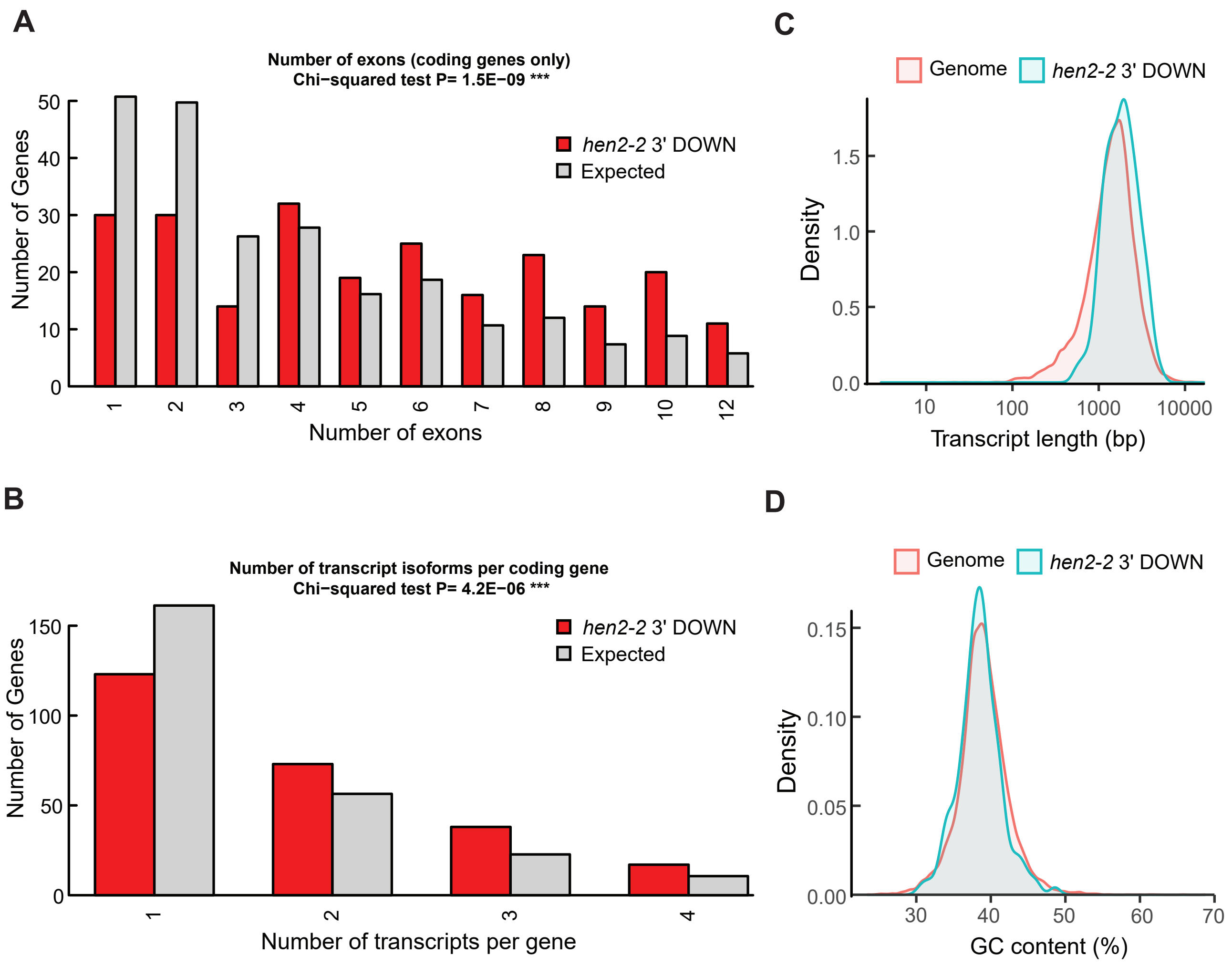

Supplementary Figure S6. Characteristics of genes down only at the 3’end in *hen2-2*.
A) Bar plot showing the number of exons in genes down at the 3'-end in *hen2-2* (n = 289). Expected scores are based on the whole Arabidopsis genome as background. B) Transcripts isoforms from 3' down genes are presented in contrast with expected number of transcripts from the whole Arabidopsis genome. C-D) Density plots depict the average transcript length and GC content of genes down at the 3'-end in *hen2-2*, respectively.

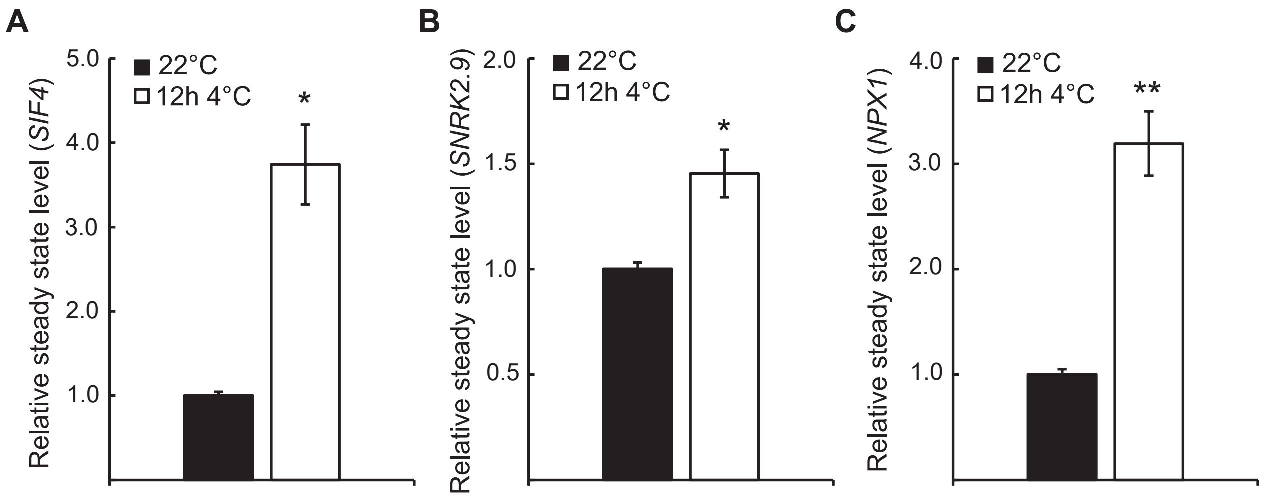

Supplementary Figure S7. RT-qPCR for novel cold responsive genes
A-C) The relative steady state level of A) *SIF4*, B) *SNRK2.9*, and C) *NPX1* measured with RT-qPCR in WT and *hen2-2* at 22°C compared to 12h at 4°C. Steady state levels were normalized to the WT levels at 22°C. The mean values are from three biological replicates. Error bars represent ± SEM. Statistical significance was calculated with Student's t-test (*p<0.05, **p<0.01).

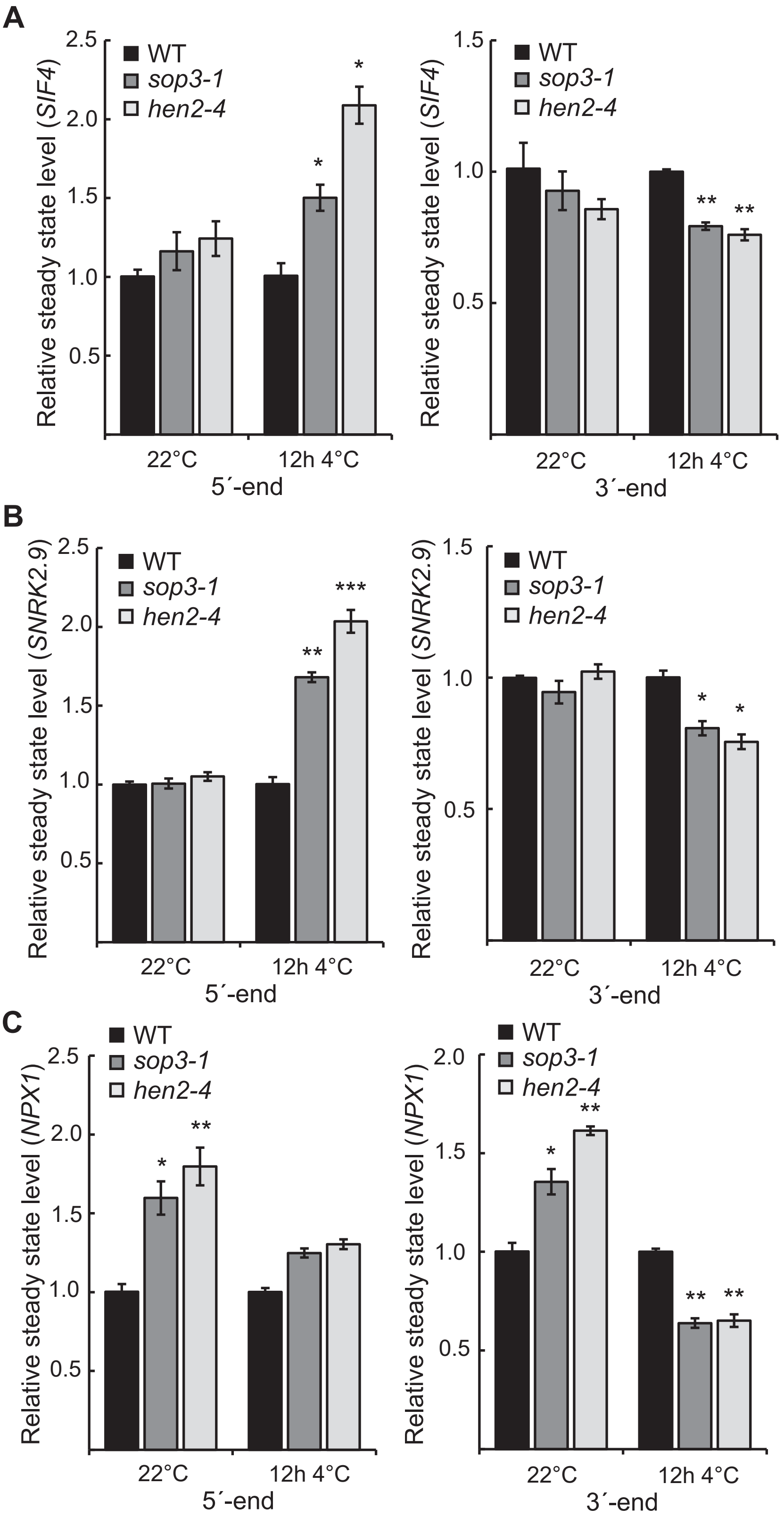

Supplementary Figure S8. RT-qPCR for cold specific transcript isoform effect with 5' and 3' specific primers on genes identified in Supplementary Figure S7 in hen2 mutant alleles.

A-C) The relative steady state level of A) *SIF4*, B) *SNRK2.9*, and C) *NPX1* measured with RT-qPCR in WT and *sop3-1* and *hen2-4* at the 5'-end (left panel) and 3'-end (right panel). Steady state levels were normalized to the WT levels at 22°C and 12h at 4°C. The mean values are from three biological replicates. Error bars represent ± SEM. Statistical significance was calculated with Student's t-test (*$p<0.05$, **$p<0.01$, ***$p<0.001$).

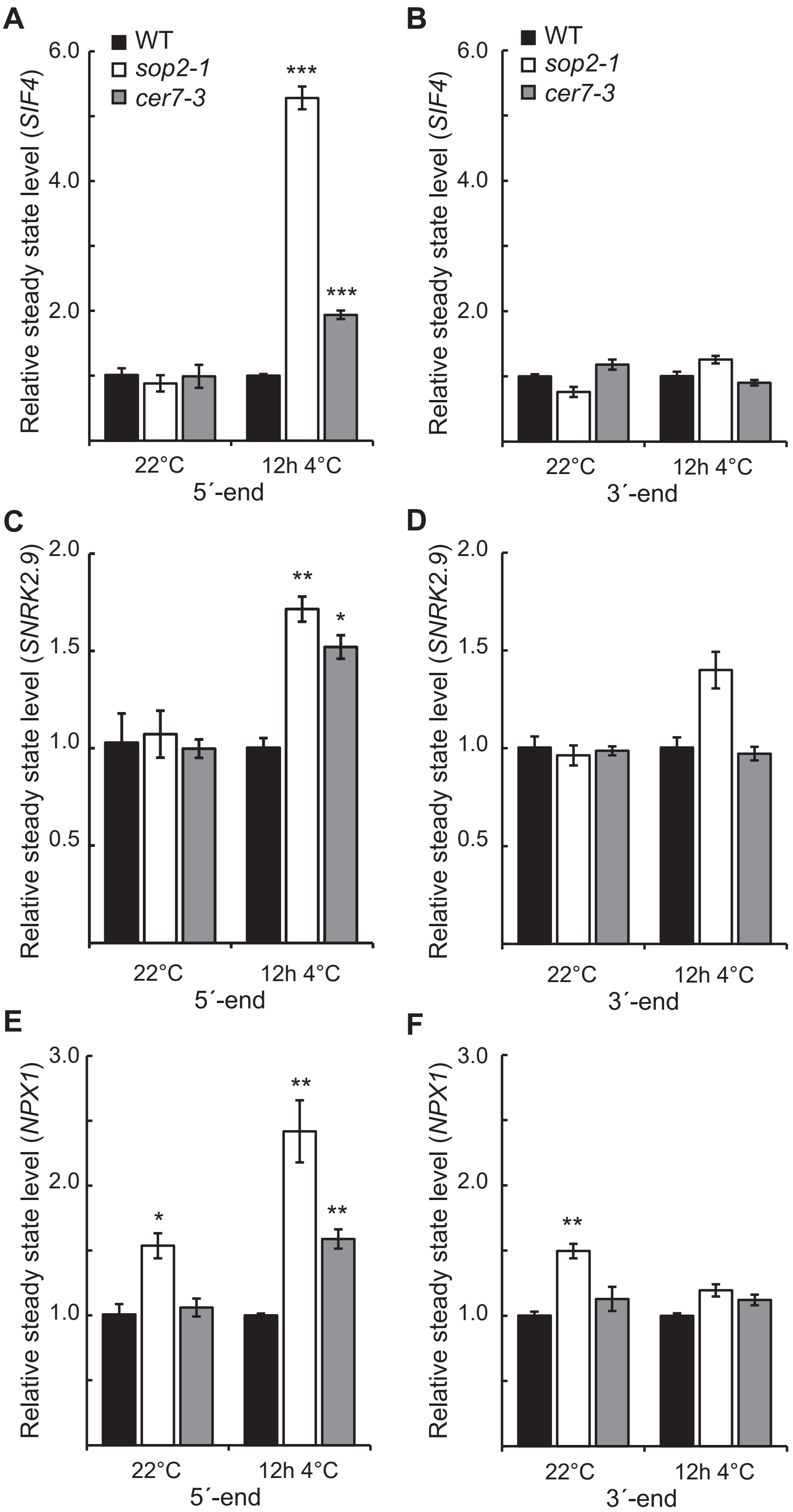

Supplementary Figure S9. RT-qPCR showing the lack cold specific transcript isoform effect with 5' and 3' specific primers on genes identified in Supplementary Figure S7 in exosome mutant alleles.
A-F) The relative steady state level of A-B) *SIF4*, C-D) *SNRK2.9*, and E-F) *NPX1* measured with RT-qPCR in WT and *sop2-1* and *cer7-3* at the 5'-end (A, C, E) and 3'-end (B, D, F). Steady state levels were normalized to the WT levels at 22°C and 12h at 4°C. The mean values are from three biological replicates. Error bars represent ± SEM. Statistical significance was calculated with Student's t-test (*p<0.05, **p<0.01, **p<0.01).
