# Supplementary material for: The nuclear exosome subunit HEN2 acts independently of the core exosome to assist transcription in Arabidopsis
Source: Plant Physiol. 2024 Sep 25;196(4):2625–37. doi: 10.1093/plphys/kiae503 (PMC11638103; doi:10.1093/plphys/kiae503)
Supplement: kiae503_Supplementary_Data [file pp.00978.2024-s06.pdf › pp.00978.2024-s06.pdf]

Plant Physiology®

The nuclear exosome subunit, HEN2, acts independently from the core exosome to assist transcription in Arabidopsis.

Susheel Bhat, Mishaneh Asgari, Sarah Mermet, Priyanka Mishra, and Peter Kindgren

Review Timeline:

Submission Date:	09-Nov-2023
Editorial Decision:	11-Dec-2023
Resubmission Received:	21-Jun-2024
Editorial Decision:	10-Jul-2024
Revision Received:	02-Aug-2024
Accepted:	26-Aug-2024

11-Dec-2023

Dr. Peter Kindgren
Umea Plant Science Centre
Forest Genetics and Plant Physiology
Umeå
Sweden

RE: The nuclear exosome subunit, HEN2, acts independently from the core exosome to stimulate transcription in Arabidopsis

Dear Dr. Kindgren:

I hope that you are well.

Thank you for giving us the opportunity to review your manuscript for publication in the Plant Physiology. The reviewers have found the manuscript of interest, but have raised a number of concerns that will require your attention. As these concerns are of some gravity and will entail substantial changes to the manuscript, you are likely to need additional time to address them. I must therefore reject the manuscript in its present form. Nonetheless, if you are able to address the points raised in review, I would welcome your resubmission of a manuscript that incorporates your responses.

I addition to addressing every comment raised by the two Reviewers, I would like to direct your attention to the following points for consideration in a resubmitted manuscript.

1. Provide evidence for the role of a second hen2 mutant allele as well as a complementing line to justify the conclusions made (not for the next generation sequencing but for all the physiology and molecular assays).

2. Monitor full-length (5' and 3') COR genes transcript abundance.

3. Provide more evidence for the specificity of the sop2-1 pleiotropic phenotype and examine the phenotypes of a zchcc8a/b double mutant to solidify the models presented.

Please note that any resubmission will be treated as a new entry to the system and is likely to go out for review.

If you do have any queries or concerns, you are welcome to contact me in the first instance.

Thank you again for allowing Plant Physiology to review your work. We look forward to hearing from you soon.

Sincerely,

Editor, Plant Physiology

--- Reviewer comments:
Reviewer #1 (Comments for the Author):

This is an interesting paper describing a new role for the nuclear exosome component HEN2. The background very well explained in the introduction for a more general reader but description of some of the results leads to ambiguity due to some of the terms used.
The story is in two parts; an observation of an altered response to cold in the mutant, which is less able to acclimate to freezing temperatures, and then a detailed analysis at the level of transcripts. The latter is significant and important but does not go far towards explaining the former. The case is well made for the effects being impossible to explain just through a defect in exosome activity.
I'd like to raise the following points:

1. A single mutant allele is used throughout. Without data from a second allele or a complementation line, linkage to this new phenotype cannot be proven.
2. The paper starts with a study of what happens to 5' and 3' ends of CBF transcripts. The most obvious question to follow this is what is the immediate consequence to accumulation of functional CBF protein, which can go onto activate cold acclimation-associated COR gene expression. To know this, transcript levels of the target COR genes should be measured and it would be sensible to include measurement of 5' and 3' ends, given the observations made so far at this stage. It sounds from the rest of the paper that these transcripts were unaffected but it is important to establish this now before measuring a reduction in freezing tolerance and speculating how it might be brought about.

3. Figure 4 is not well explained in the manuscript. It would be important to highlight here that they are using the RNAseq data to assess complete/partial transcripts and to state explicitly whether this can or cannot be done with no bias to the 3' or 5' end. This issue is addressed later in lines 226-228 with a refinement to the protocol to deal with it but it is important for the reader to know this before attempting to make sense of Fig 4 data.
4. Line 263-4. Why would you expect a higher level of RNAP engagement at the 5' end of genes in the mutant. As far as I can see, there is no suggested reason for the increased initial transcript levels (at the 5' end) of the CBF genes in the hen2 mutant. Given the proposed effects of HEN2, it would have been useful to see ChIP or planet data for the CBF genes. Is there more transcription starting (and not completing) or are the short transcripts more stable because they lack a regulatory region at the 3' end? Transcript stability of the CBFs is governed by temperature. The manuscript does not really provide a satisfactory explanation for this part of the phenomenon.
5. Given so many transcript are NOT affected by loss of HEN2 (including, presumably, those of the COR genes), it would be helpful to see further evidence of what makes a transcript susceptible. Are these highly upregulated genes or moderate up regulated ones? Does the phenotype have anything to do with how much transcription is needed quickly, the length of the transcript, whether it has introns or not (the CBFs do not)?
Other points:
I don't entirely agree that abiotic stress looks like one of the main GO terms coming up in the mutant DE genes under cold. RNA processing clearly does but no response to cold or major abiotic stress gene groups. See also comment 2 above. Are supp tables 3 and 4 ALL DE genes (both up and down)? Would be useful to see both separately.

I don't follow the logic of the interpretation for the biomass experiments. The mutant already has lower biomass so it cannot be seen as cold-specific, even though there is a larger differential at 10 degrees. Why choose this temperature? It is not the acclimation temperature. I think it would be difficult to say this is part of a cold-sensitive phenotype and there is no clear reason why this would be related to the regulation of the CBF genes. In fact, active CBFs in the cold would repress growth through their action on the DELLA pathway.

Figure 1B. Why are there only 2 biological replicates? The significance of the differences cannot be tested. However, parts C to D do show significant increases in the abundance of the 5' end of the CBF transcripts with a reduction in the abundance of the 3' ends.
Please explain how planet differs from chip. It is still measuring RNAPII occupancy rather than transcription per se?

Lines 277-278 this is a hypothesis but cannot make it sounds like the results indicate this.

Lines 329-330. "increasing evidence" needs more than one reference and something more recent than 2006.

Reviewer #2 (Comments for the Author):

Bhat et al. report that the Arabidopsis RNA helicase HEN2 stimulates RNAPII activity at cold-responsive loci and that this role is independent of its function in exosome-mediated RNA degradation. This manuscript is definitely of interest but we think that the conclusions must be further supported as recommended below. A first caveat of the manuscript is that the HEN2-mediated stimulation of RNAPII transcription elongation is not directly addressed, and that alternative possibilities exist to explain the data. Second, it remains to be formally demonstrated that the proposed HEN2 function is independent of the exosome or the NEXT complex. Finally, it would interesting that the proposed regulation of the C-repeat binding factor (CBF) loci by HEN2-mediated stimulation of RNAPII transcription elongation in cold is discussed together with the other processes reported to regulate these loci.

Major comments
1- The conclusion about a function of HEN2 independently of the RNA exosome is a strong and new claim. Therefore, it must be demonstrated. sop2-1 is a hypomorphic mutant that may affect only a subset of exosome functions, or/and has only mild affects. If a molecular phenotype is observed in sop2-1, it is perfectly fine to use this mutant to assign a molecular role to the RNA exosome, as the authors have done in previous studies. However, we wonder whether this mutant is suitable to conclude about the absence of involvement of the RNA exosome based on a negative result. The authors should test both the cold sensitivity and the differential signals of 5'/3' regions for the 6 candidate mRNAs in amirRRP4, amirRRP44 and/or cer3-7 lines. These mutants are also not null-mutants, but they are more strongly affected than the sop2-1, and similar observations in independent mutants are required to strengthen their point.

2- In a similar way, single zchcc8 mutants cannot be fully expected to show an effect, and therefore the double zchcc8a/b must be tested to rule out the involvement of NEXT. In case NEXT is not involved, the authors may want to discuss that some RNA substrates could be degraded by the help of other exosome targeting complexes such as PAXT.

3- Despite the title "hen2-2 functions independent of known interaction proteins to regulate the CBFs", no hen2 data are shown in Figure 3. Was the hen2-2 mutant analyzed together with the other exosome mutants? If this was indeed the case, presenting the data altogether will strengthen the conclusion.

4- The PI and his current and past colleagues have previously shown that cold acclimatation in Arabidopsis involves the regulation of the C-repeat binding factor 1 (CBF1) locus by RNAPII collision due to the transcription a long non-coding RNA transcribed in antisense orientation, and by RNA silencing triggered by double strand RNA formation of the cis-natural antisense transcript and CBF1 mRNA (https://doi.org/10.1016/j.xplc.2023.100551; https://doi.org/10.1038/s41467-018-07010-6). The proposed impact of HEN2 on RNAPII transcription elongation must be discussed alongside the two other types of regulation at the CBF1 locus.

5- The authors show detailed RNA-seq signals for several genes but not for CBF1-3. Please show and discuss the RNA-seq signals detected for CBF1-3 in the RNA-seq experiments. Are they coherent with the qPCR experiments?

6- The authors should discuss the issue of using oligo(dT) purified RNA for the RNA-seq experiments and refer to previous work by the Marquardt lab. First, readers may not understand that the 5' transcripts are detected as poly(A)+ RNAs. Second, what about the possibility that non-polyadenylated RNAs are not detected by RNA-seq at these loci?

7- Line 174, "The phenotype can partly be explained by its mis-regulation of the CBF genes". This speculation is not addressed here, and should not be presented as a conclusion in the results. Are the primary target genes of CBF mis-regulated in hen2?

8- Line 176 "...indicate a co-transcriptional role for HEN2...". Both qRT PCR and RNA seq measure RNA steady-state levels and do not discriminate between transcriptional and post-transcriptional effects.

9- In fact, the experiments in this manuscript do not really address stimulation of transcription. There are other possible explanations. For example, HEN2 may be needed to degrade a non-coding RNA that otherwise stimulates premature transcription termination or alternative PAS choice.

10- Line 296 "HEN2 interacts with the cap-binding complex (CBC) that puts the cap on the 5´-end of newly synthesized RNA exiting RNAPII (Lange et al., 2014). This interaction provides a direct coupling between the 3'-end processing roles of HEN2 and the 5'-capping of the nascent RNA by the CBC." First, CBC binds to the cap, but it does not catalyze 5' capping. Second, in analogy to mammalian cells, the recruitment of HEN2 to nascent transcripts would perhaps stimulate promotor proximal termination and degradation by the exosome. This fits to the accumulation of short transcripts that are detected with the 5' primer pairs in fig 1B. However, "3' processing roles" in the sense of an active participation to cleavage and polyadenylation of mRNAs have not been demonstrated in mammals or plants.

11- "The nuclear speckles are similar to those where active transcription occurs". We do not understand this statement, because those speckles could look similar but could be completely unrelated. Have RNAPII and HEN2 been detected in the same speckles?

12- Fig. 7D: We guess that the statistical significance was calculated between WT and hen2-2? Is the difference significant between hen2 12h 4{degree sign}C 5' end and hen2 12h 4{degree sign}C 3' end in 7B + C? Also, is the level of expression of these transcripts sufficient in the reference conditions (22{degree sign}C, WT) to not overestimate the fold-change values?

Minor comments
13- Please include the legends in the supplemental figures.

14- We suggest that FC /adjp values, annotation or at least bio-type info, etc.. are added to the AGI provided as tables S1 and S2.

15- The color code in Fig 4 B, C, D should be homogenized.

Response to reviewers

From editor:

1. Provide evidence for the role of a second hen2 mutant allele as well as a complementing line to justify the conclusions made (not for the next generation sequencing but for all the physiology and molecular assays).

Thank you for these suggestions. We have added results from two other HEN2 mutant alleles (*sop3-1* and *hen2-4*) to the manuscript. Both mutants have similar transcriptional and cold acclimation defects as *hen2-2*. In addition, we have added data from a complementation line of *hen2-2* (35S:*HEN2*). The complemented line rescues both the transcriptional and cold acclimation defects of *hen2-2*.

2. Monitor full-length (5' and 3') COR genes transcript abundance.

To address this comment, we have added Supplemental Figure S4. This new figure shows the RNA levels of all *COR* genes as well as the targets of *CBF1-3*. In all targets of the CBFs, there is a decreased RNA-seq signal specifically at the 3'-end. This means that the *hen2-2* mutant has lower levels of full length, cold-induced mRNAs which better explain the cold sensitivity phenotype of the *HEN2* mutants.

3. Provide more evidence for the specificity of the sop2-1 pleiotropic phenotype and examine the phenotypes of a zchcc8a/b double mutant to solidify the models presented.

We have added data from the *zchcc8a/b* double mutant and the *cer7-3* mutant. The *cer7-3* mutant corroborates our earlier results from the *sop2-1* mutant. The *zchcc8a/b* double mutant show a mis-regulation of the *CBFs*, but not an accumulation of 5'-end isoforms, thus not in the same degradation pathway as HEN2. Although this is an interesting finding, we believe further experiments are outside the scope of this manuscript.

Reviewer #1 (Comments for the Author):

This is an interesting paper describing a new role for the nuclear exosome component HEN2. The background very well explained in the introduction for a more general reader but description of some of the results leads to ambiguity due to some of the terms used.

The story is in two parts; an observation of an altered response to cold in the mutant, which is less able to acclimate to freezing temperatures, and then a detailed analysis at the level of transcripts. The latter is significant and important but does not go far towards explaining the former. The case is well made for the effects being impossible to explain just through a defect in exosome activity. I'd like to raise the following points:

1. A single mutant allele is used throughout. Without data from a second allele or a complementation line, linkage to this new phenotype cannot be proven.

Thank you for these suggestions that have enhanced the manuscript greatly. We have added two more *hen2* alleles, another core subunit mutant of the nuclear exosome (*cer7-3*) and a complemented line of *hen2-2* (35:*HEN2*). The new results corroborate our model.

2. The paper starts with a study of what happens to 5' and 3' ends of CBF transcripts. The most obvious question to follow this is what the immediate consequence to accumulation of functional CBF protein is, which can go onto activate cold acclimation- associated COR gene expression. To know this, transcript levels of the target COR genes should be measured, and it would be sensible to include measurement of 5' and 3' ends, given the observations made so far at this stage. It sounds from the rest of the paper that these transcripts were unaffected, but it is important to establish this now before measuring a reduction in freezing tolerance and speculating how it might be brought about.

We agree, this is a point we did not fully elaborate on in the previous version of the manuscript. In the resubmitted manuscript, we have added Supplemental Figure S4. The new figure shows the RNA-seq profile of *COR* genes as well as the targets of the individual CBFs. We believe there are two main points to consider here. Firstly, in general, both *COR* genes and CBF targets are mis-regulated at control conditions in the *hen2-2* mutant. They will therefore be disregarded in our further analysis as they are not specifically mis-regulated after cold stress in *hen2* mutants. However, since the *CBFs* are downregulated after cold stress in *hen2* mutants, many *COR* genes are DOWN after cold which further explains the cold sensitivity of *hen2* mutants (see example *COR78*, a well-studied *COR* gene below that highlight this point).

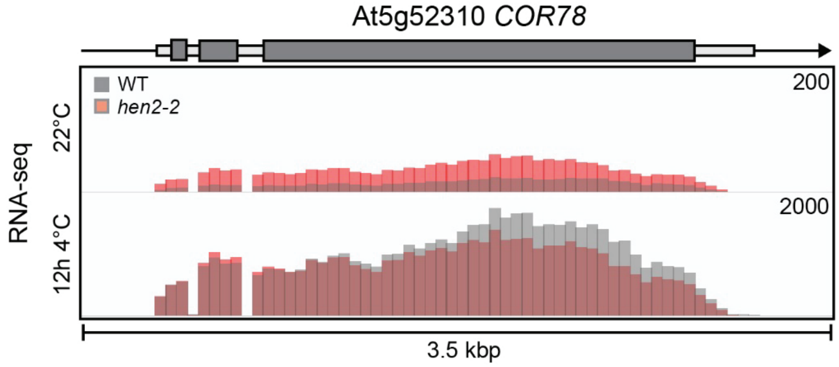

Figure 1. *Screenshot of overlayed RNA-seq data at 22°C and 12h at 4°C from wild type (grey) and hen2-2 (red) of COR78 (At5g52310)*

Secondly, in all CBF targets, we see a specific decrease of RNA-seq signal at the 3'-end of genes, suggesting that some cold-induced genes are HEN2 dependent during cold stress (e.g. our candidate genes in Figure 6-7) and some are mis-regulated due to lower *CBF* levels in *hen2* mutants (e.g. *COR28* in Supplemental Figure S3C). We have summarized these points in an elaborating text in the manuscript.

3. Figure 4 is not well explained in the manuscript. It would be important to highlight here that they are using the RNAseq data to assess complete/partial transcripts and to state explicitly whether this can or cannot be done with no bias to the 3' or 5' end. This issue is addressed later in lines 226-228 with a refinement to the protocol to deal with it but it is important for the reader to know this before attempting to make sense of Fig 4 data.

Thank you for this comment. We have tried to make this clearer in the resubmitted manuscript. The results in Figure 4 and the new Supplemental Figure S4 are a prerequisite for the refined analysis in Figure 5. We believe that presenting the results this way gives a logical sequence to our reasoning.

4. Line 263-4. Why would you expect a higher level of RNAP engagement at the 5' end of genes in the mutant. As far as I can see, there is no suggested reason for the increased initial transcript levels (at the 5' end) of the CBF genes in the hen2 mutant. Given the proposed effects of HEN2, it would have

been useful to see ChIP or planet data for the CBF genes. Is there more transcription starting (and not completing) or are the short transcripts more stable because they lack a regulatory region at the 3' end? Transcript stability of the CBFs is governed by temperature. The manuscript does not really provide a satisfactory explanation for this part of the phenomenon.

A higher RNAPII occupancy at 5′-end of genes would be one direct explanation of why *hen2* mutants accumulate 5′-end isoforms. However, this is not what we observe. In Figure 7, we show that we detect wild type-like levels of RNA associated with RNAPII for our candidate genes at the 5′-end. This strongly suggests that transcription initiation of the candidate genes is not an issue in *hen2-2*. These observations are important to disentangle any initiation from elongation roles for HEN2. The short isoforms we see is only detected in *hen2* and nuclear exosome mutants, indicating that these are rapidly degraded in wild type (i.e. highly unstable). Thus, transcript stability for these transcripts is not mainly dictated by temperature but rather their degradation pattern. This holds true for the 5′-end isoforms of the *CBFs* as well that we detect in *hen2* mutants. We do not show that HEN2 is involved in the elongation of the *CBFs*, but rather for a set of cold-induced genes after 12h at 4°C. Together, the mis-regulation of the *CBFs* and the elongation role of HEN2 at our candidate genes explains the cold sensitivity in *hen2* mutants. We have made this point clearer in the manuscript.

5. Given so many transcripts are NOT affected by loss of HEN2 (including, presumably, those of the COR genes), it would be helpful to see further evidence of what makes a transcript susceptible. Are these highly upregulated genes or moderate up regulated ones? Does the phenotype have anything to do with how much transcription is needed quickly, the length of the transcript, whether it has introns or not (the CBFs do not)?

Thank you for this suggestion. We have added Supplemental Figure S5 in response to this comment. The genes specifically DOWN in the 3′-end at 12h 4°C in *hen2-2* tend to be cold-induced (Figure 5C), multi-exonic and have multiple isoforms (Supplemental Figure 5A-B), and longer than the genomic median (Supplemental Figure 5C). Part of the explanation for these results is most probably a technical effect of our analysis. Longer genes would have a higher probability to be caught in our analysis due to statistical power of the RNA-seq signal along a gene. But this does not fully explain the results. The correlation between splicing, RNAPII stalling around splice sites (the major intragenic stalling site for RNAPII), and *hen2-2* DE genes may suggest that splicing is a determinant of premature termination, and this could be a HEN2 dependent point for continued elongation. We believe this is speculative at this point and have avoided mentioning it in the text, but a clear avenue for future research.

Other points:

I don't entirely agree that abiotic stress looks like one of the main GO terms coming up in the mutant DE genes under cold. RNA processing clearly does but no response to cold or major abiotic stress gene groups. See also comment 2 above. Are supp tables 3 and 4 ALL DE genes (both up and down)? Would be useful to see both separately.

Thank you for this suggestion. In the resubmitted manuscript, we have used PANTHER for the GO-analysis and divided the genes UP/DOWN at the different temperatures. We agree with the reviewer that this is a better way to illustrate the mis-regulation of genes in *hen2-2*. The new analysis shows that cold responsive genes are DOWN at both 22°C and 12h 4°C. Overall, many stress-responsive genes and processes affected by temperature (e.g. photosynthesis) are mis-regulated in *hen2-2*.

I don't follow the logic of the interpretation for the biomass experiments. The mutant already has lower biomass so it cannot be seen as cold-specific, even though there is a larger differential at 10

degrees. Why choose this temperature? It is not the acclimation temperature. I think it would be difficult to say this is part of a cold-sensitive phenotype and there is no clear reason why this would be related to the regulation of the CBF genes. In fact, active CBFs in the cold would repress growth through their action on the DELLA pathway.

Point taken and we agree. We have removed the biomass experiment that might be confusing for the reader. Instead, to strengthen our point of *hen2* mutants being cold sensitive, we have added two more mutant alleles of *HEN2* and a complemented line. The freezing tolerance and transcriptional data corroborate our earlier conclusions.

Figure 1B. Why are there only 2 biological replicates? The significance of the differences cannot be tested. However, parts C to D do show significant increases in the abundance of the 5' end of the CBF transcripts with a reduction in the abundance of the 3' ends. Please explain how planet differs from chip. It is still measuring RNAPII occupancy rather than transcription per se?

Figure 1A-B is a visualization of Transcription Start Site-sequencing. This data comes from available resources (PMID: 32444691). The TSS-seq was done with 2 biological replicates. Figure 1C-D is RT-qPCR data from independent samples (3 biological replicates) collected for this study.

In this study, we have used plaNET-qPCR (Figure 7) to isolate actively transcribed RNA (prior to any degradation) to disentangle the transcriptional role of HEN2 at our candidate genes. The genome-wide technique, plaNET-seq, detects actively transcribing RNAPII complexes in a strand-specific and to single nucleotide resolution. ChIP-seq on the other hand, detects binding to DNA (i.e. not strand-specific) and the resolution is dependent on the fragmentation of the isolated DNA.

Lines 277-278 this is a hypothesis but cannot make it sounds like the results indicate this.

We have removed this sentence to avoid any confusion.

Lines 329-330. "increasing evidence" needs more than one reference and something more recent than 2006.

Agreed. We have added more references to support our statement.

Reviewer #2 (Comments for the Author):

Bhat et al. report that the Arabidopsis RNA helicase HEN2 stimulates RNAPII activity at cold-responsive loci and that this role is independent of its function in exosome-mediated RNA degradation. This manuscript is definitely of interest, but we think that the conclusions must be further supported as recommended below. A first caveat of the manuscript is that the HEN2-mediated stimulation of RNAPII transcription elongation is not directly addressed, and that alternative possibilities exist to explain the data. Second, it remains to be formally demonstrated that the proposed HEN2 function is independent of the exosome or the NEXT complex. Finally, it would interesting that the proposed regulation of the C-repeat binding factor (CBF) loci by HEN2-mediated stimulation of RNAPII transcription elongation in cold is discussed together with the other processes reported to regulate these loci.

Major comments:

1- The conclusion about a function of HEN2 independently of the RNA exosome is a strong and new claim. Therefore, it must be demonstrated. sop2-1 is a hypomorphic mutant that may affect only a

subset of exosome functions, or/and has only mild affects. If a molecular phenotype is observed in sop2-1, it is perfectly fine to use this mutant to assign a molecular role to the RNA exosome, as the authors have done in previous studies. However, we wonder whether this mutant is suitable to conclude about the absence of involvement of the RNA exosome based on a negative result. The authors should test both the cold sensitivity and the differential signals of 5'/3' regions for the 6 candidate mRNAs in amirRRP4, amirRRP44 and/or cer3-7 lines. These mutants are also not null-mutants, but they are more strongly affected than the sop2-1, and similar observations in independent mutants are required to strengthen their point.

We thank the reviewer for this suggestion. We have added the *cer7-3* mutant to Figure 3 and Supplemental Figure 8. The *cer7-3* mutant show similar defects as *sop2-1*.

2- In a similar way, single zchcc8 mutants cannot be fully expected to show an effect, and therefore the double zchcc8a/b must be tested to rule out the involvement of NEXT. In case NEXT is not involved, the authors may want to discuss that some RNA substrates could be degraded by the help of other exosome targeting complexes such as PAXT.

Agreed, we have added the double mutant to the resubmitted manuscript (Figure 3). We do see a lower induction of the *CBFs* in the *zchcc8* double mutant in the 3'-end but no difference to wild type in the 5'-end. This suggests that ZCHCC8 may be involved in the regulation of the *CBFs* but independent of RBM7 and the core nuclear exosome. That ZCHCC8 seems to work independently of its known interaction partners is interesting to follow up in future projects.

Our results clearly show that the NEXT complex is not involved in the degradation of 5'-end isoforms of the CBFs, therefore we have added a section about alternative guiding complexes (including PAXT) to enhance the discussion. We thank the reviewer for the suggestion.

3- Despite the title "hen2-2 functions independent of known interaction proteins to regulate the CBFs", no hen2 data are shown in Figure 3. Was the hen2-2 mutant analyzed together with the other exosome mutants? If this was indeed the case, presenting the data altogether will strengthen the conclusion.

Yes, *hen2-2* was analyzed with the other mutants, and we have added this data to Figure 3.

4- The PI and his current and past colleagues have previously shown that cold acclimatation in Arabidopsis involves the regulation of the C-repeat binding factor 1 (CBF1) locus by RNAPII collision due to the transcription a long non-coding RNA transcribed in antisense orientation, and by RNA silencing triggered by double strand RNA formation of the cis-natural antisense transcript and CBF1 mRNA (https://doi.org/10.1016/j.xplc.2023.100551; https://doi.org/10.1038/s41467-018-07010-6). The proposed impact of HEN2 on RNAPII transcription elongation must be discussed alongside the two other types of regulation at the CBF1 locus.

Thank you for highlighting our earlier research and the suggestion. We have added text to the introduction and discussion concerning the regulation of the *CBFs*.

5- The authors show detailed RNA-seq signals for several genes but not for CBF1-3. Please show and discuss the RNA-seq signals detected for CBF1-3 in the RNA-seq experiments. Are they coherent with the qPCR experiments?

The *CBF* genes peak their expression after around 3h at 4°C and all *CBF* data in the manuscript is from this time point. The RNA-seq data we have is from 12h at 4°C when the *CBFs* have peaked and started to be repressed (e.g. by the lncRNA *SVALKA* in the *CBF1* context). We do not claim that HEN2

is involved in the elongation of the *CBFs,* and it would therefore be misleading to show the RNA-seq for the *CBFs* at 12h 4°C when other regulatory mechanisms are active.

6- The authors should discuss the issue of using oligo(dT) purified RNA for the RNA-seq experiments and refer to previous work by the Marquardt lab. First, readers may not understand that the 5' transcripts are detected as poly(A)+ RNAs. Second, what about the possibility that non-polyadenylated RNAs are not detected by RNA-seq at these loci?

We have added more information in the text that clearly states we have used a poly(A)+ RNA for the RNA-seq experiment so readers understand this (in methods and results part). While we might underestimate the level of non-polyadenylated RNAs in the RNA-seq experiment, we confirm the results at the candidate genes with RT-qPCR. The is cDNA synthesized by random primers and/or gene specific primers that produce cDNA from both poly(A) RNAs and non-polyadenylated RNAs. We have also added a paragraph in the discussion about polyadenylation of these transcripts and potential consequences, referring to our and earlier reports.

7- Line 174, "The phenotype can partly be explained by its mis-regulation of the CBF genes". This speculation is not addressed here and should not be presented as a conclusion in the results. Are the primary target genes of CBF mis-regulated in hen2?

Agreed, we have removed this sentence from the manuscript and come back to it when presenting the results of the new Supplementary Figure S4.

8- Line 176 "...indicate a co-transcriptional role for HEN2...". Both qRT PCR and RNA seq measure RNA steady-state levels and do not discriminate between transcriptional and post-transcriptional effects.

Thanks for pointing this out, it is misleading to put that statement here. We have reformulated this sentence to better conclude the results obtained at this stage.

9- In fact, the experiments in this manuscript do not really address stimulation of transcription. There are other possible explanations. For example, HEN2 may be needed to degrade a non-coding RNA that otherwise stimulates premature transcription termination or alternative PAS choice.

Agreed, "stimulate" is a poorly chosen word that does not reflect our data. We have changed the word "stimulate" to "assist" in the title and in the text to better encompass our findings. We find it unlikely that any non-coding RNA would be responsible for the effects we see in *hen2* mutants since we should then see a similar effect in other exosome mutants. However, we agree that other mechanisms are possible, hence the change in wording.

10- Line 296 "HEN2 interacts with the cap-binding complex (CBC) that puts the cap on the 5′-end of newly synthesized RNA exiting RNAPII (Lange et al., 2014). This interaction provides a direct coupling between the 3'-end processing roles of HEN2 and the 5'-capping of the nascent RNA by the CBC." First, CBC binds to the cap, but it does not catalyze 5' capping. Second, in analogy to mammalian cells, the recruitment of HEN2 to nascent transcripts would perhaps stimulate promotor proximal termination and degradation by the exosome. This fits to the accumulation of short transcripts that are detected with the 5' primer pairs in fig 1B. However, "3' processing roles" in the sense of an active participation to cleavage and polyadenylation of mRNAs have not been demonstrated in mammals or plants.

We have revised the sentence to better reflect the literature and our findings.

11- "The nuclear speckles are similar to those where active transcription occurs". We do not understand this statement, because those speckles could look similar but could be completely unrelated. Have RNAPII and HEN2 been detected in the same speckles?

We agree that this was a bit speculative. We have removed this sentence from the text.

12- Fig. 7D: We guess that the statistical significance was calculated between WT and hen2-2? Is the difference significant between hen2 12h 4{degree sign}C 5' end and hen2 12h 4{degree sign}C 3' end in 7B + C? Also, is the level of expression of these transcripts sufficient in the reference conditions (22{degree sign}C, WT) to not overestimate the fold-change values?

Yes, the statistical significance was calculated between WT and *hen2-2*. We believe that this is the best way to show the data since these genes are cold-induced and we want the reader to focus on the difference between wild-type and *hen2-2* at different temperatures. The Ct values from investigated genes were all less than 33 and stable between biological replicates, indicating a robust result.

Minor comments:

13- Please include the legends in the supplemental figures.

Done

14- We suggest that FC /adjp values, annotation or at least bio-type info, etc.. are added to the AGI provided as tables S1 and S2.

Done

15- The color code in Fig 4 B, C, D should be homogenized.

We are reluctant to change the colour code in Figure 4A and B since they show different things. We have changed the colour code in Figure 4C-D to be homogenised to Figure 4B.

10-Jul-2024

Dr. Peter Kindgren
Umea Plant Science Centre
Forest Genetics and Plant Physiology
Umeå
Sweden

RE: The nuclear exosome subunit, HEN2, acts independently from the core exosome to assist transcription in Arabidopsis.

Dear Dr. Peter Kindgren:

We are pleased to accept your manuscript for publication in the Plant Physiology.
This acceptance is contingent on revision based on the comments of our reviewers.

In particular, please make the relevant changes suggested by Reviewer 2 (points 1-7) and if possible address points 8 and 9 .

Please highlight all changes and include a detailed annotation to changes to the text, with line numbers, and noting your responses to the comments.

To submit your revised manuscript, click:

Link Not Available

If you cannot return the revised manuscript within 8 weeks of receipt, please let us know. Otherwise, we will assume that you have elected not to revise the manuscript and are withdrawing it.

Thank you for allowing us to review your work. We look forward to hearing from you soon.

Sincerely,

Editor, Plant Physiology

-- Reviewer comments:
Reviewer #1 (Comments for the Author):

I thank the reviewers for addressing all of my queries. The new manuscript is greatly strengthened by the inclusion of new data to support linkage of the phenotypes to the hen2 locus. The new supplementary figures are also successful in addressing specific gaps I mentioned.

Reviewer #2 (Comments for the Author):

We thank the authors for addressing our comments. The authors have analyzed additional lines (different hen2 and exosome mutant alleles, as well as HEN2 complemented line) and these new data strengthen their study. We have only minor comments that the authors may want to consider.

1. Line 433 "In conclusion, we propose a novel role for the nuclear exosome subunit HEN2 that is independent of its exosome role". We suggest to rephrase this sentence and not describe HEN2 as a nuclear exosome subunit, but rather as a factor assisting the nuclear RNA exosome.
2. Lines 74-75. We have already pointed in our previous review that the CBC does not catalyze 5' capping. I think that "cap-binding complex" is self-explanatory. In case the authors insist on this sentence please replace "puts" by "binds" in the following sentence "HEN2 interacts with the cap-binding complex (CBC) that puts the cap on the 5´-end of newly synthesized RNA exiting RNAPII".
3. In the text and in Fig 1, we are a bit puzzled by the use of "qPCR probe". May be region probed by qPCR?
4. Fig S4. We guess that the shaded areas represent the variability between standard deviations for the genes considered. If this assumption is right, does the claim lines 265-7 stands? : "When plotting all COR genes, we could detect a tendency in hen2-2, earlier seen in the CBF genes with higher RNA level in the 5′-end and lower level in the 3′-end (Supplemental Figure S4A)". It seems that the difference in the mean is still within the difference in standard deviations? Same for CBF targets. Please better explain Fig S4 and clarify tendency.
5. Fig S4 We apologise but we do not understand the peculiar drop of z-score for CBF targets (panels B,C and D).

6. We recommend that the authors include a figure, e.g. a scatterplot, that compare the levels of each AGI in hen2 vs WT for each of the conditions to better support the conclusion line 271 : Taken together, the misregulation of the CBFs and "the overall lower mRNA level of genes targeted by the CBFs" give a compelling reason of why hen2-2 is cold sensitive.
7. Line 378, we appreciate that the authors added a few lines to integrate their previous findings. However, a deeper discussion as of why the previously observed stabilisation of svalka in hen2 mutants at 4{degree sign}C, which was shown to abrogate CBF transcription, is not important for the cold sensitive phenotype of hen2 mutants and/or does or does not indirectly affect the decreased full-length transcription at CBF target loci would be certainly of interest to the readers.

The following suggestions were not made during the first round of review. It is left to the authors to decide whether they want to perform such controls.
8. It would have been interesting to include a known substrate of NEXT in the analysis presented in Fig. 3A.
9. Fig 2, S1. It would be interesting to understand whether the cold-sensitivity is indeed linked to the decrease in 3' transcription of CBFs (or perhaps the upregulation of 5' regions). Do exosome mutants or ZCHCC8A/B share the cold-sensitive phenotype?

Response to reviewers (PP2024-RA-00978D)

Reviewer #2 (Comments for the Author):

We thank the authors for addressing our comments. The authors have analyzed additional lines (different hen2 and exosome mutant alleles, as well as HEN2 complemented line) and these new data strengthen their study. We have only minor comments that the authors may want to consider.

1. Line 433 "In conclusion, we propose a novel role for the nuclear exosome subunit HEN2 that is independent of its exosome role". We suggest to rephrase this sentence and not describe HEN2 as a nuclear exosome subunit, but rather as a factor assisting the nuclear RNA exosome.

Agreed, we have rephrased this sentence (Line number: 391)

2. Lines 74-75. We have already pointed in our previous review that the CBC does not catalyze 5' capping. I think that "cap-binding complex" is self-explanatory. In case the authors insist on this sentence please replace "puts" by "binds" in the following sentence "HEN2 interacts with the cap-binding complex (CBC) that puts the cap on the 5 ́-end of newly synthesized RNA exiting RNAPII".

We have replaced "puts" to "binds to" in the mentioned sentence (Line number: 66)

3. In the text and in Fig 1, we are a bit puzzled by the use of "qPCR probe". May be region probed by qPCR?

To avoid confusion, we show the region amplified in the RT-qPCR as two oligos and changed "qPCR probe" to RT-qPCR oligos" in the figures and changed the text accordingly.

4. Fig S4. We guess that the shaded areas represent the variability between standard deviations for the genes considered. If this assumption is right, does the claim lines 265-7 stands? "When plotting all COR genes, we could detect a tendency in hen2-2, earlier seen in the CBF genes with higher RNA level in the 5'-end and lower level in the 3'-end (Supplemental Figure S4A)". It seems that the difference in the mean is still within the difference in standard deviations? Same for CBF targets. Please better explain Fig S4 and clarify tendency.

The data used in Figure S4 was obtained from the publication Song et al., 2012. 146 COR genes that are direct targets of CBF1, CBF2 and CBF3 were identified in this study. Since 146 is not a huge number of genes, their biological variance is much more apparent when making metaplots. In our bid to be as comprehensive as possible we decided to include the standard deviation in our plots, which is not a very common practice. Below is the plot without the standard deviation shaded in. We agree that with the shaded standard deviation the data looks less convincing. This is the reason why we have this data as a supplementary figure and in text we used the word 'tendency' with utmost care.

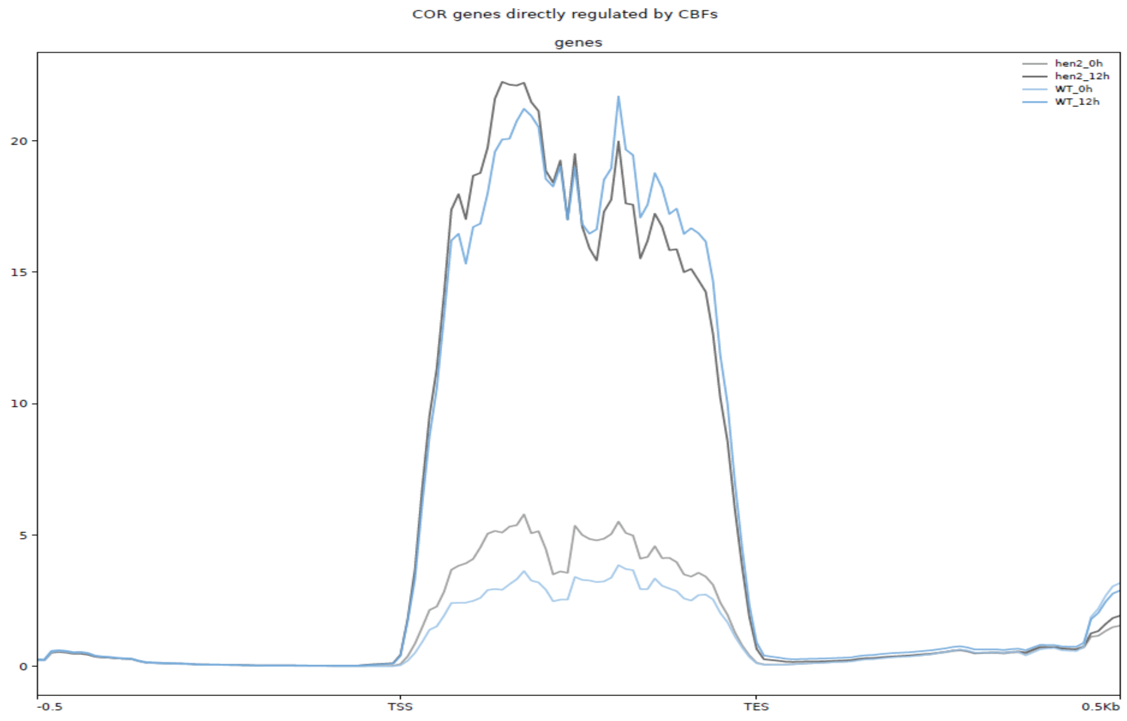

5. Fig S4 We apologise but we do not understand the peculiar drop of z-score for CBF targets (panels B,C and D).

We thank the reviewer for this very keen observation. We looked into our pipeline and identified that there was a mismatch in the reference genome used to generate the bigwigs (Araport11) with that used for generating the gtfs for our target genes (TAIR10). We have now unified this all to Araport11. Only Fig S4 B, C and D were affected by this mismatch and those graphs have been updated.

6. We recommend that the authors include a figure, e.g. a scatterplot, that compare the levels of each AGI in hen2 vs WT for each of the conditions to better support the conclusion line 271 : Taken together, the misregulation of the CBFs and "the overall lower mRNA level of genes targeted by the CBFs" give a compelling reason of why hen2-2 is cold sensitive.

Thank you for this suggestion. We have chosen to add scatterplots for all DE genes (new Supplementary Figure 4) to keep the flow of the text. The new figure clearly show that many genes are lower expressed in* hen2-2 *at both time points. We have also rephrased the last sentence in the paragraph to reflect these results (Line number 247).

7. Line 378, we appreciate that the authors added a few lines to integrate their previous findings. However, a deeper discussion as of why the previously observed stabilisation of svalka in hen2 mutants at 4{degree sign}C, which was shown to abrogate CBF transcription, is not important for the cold sensitive phenotype of hen2 mutants and/or does or does not indirectly affect the decreased full-length transcription at CBF target loci would be certainly of interest to the readers.

We have added more to the discussion concerning SVALKA and hen2 (Line number 343-347). It is important to point out that SVALKA is not stabilized in* hen2 *mutants, rather the read-though transcript of SVALKA (asCBF1).

The following suggestions were not made during the first round of review. It is left to the authors to decide whether they want to perform such controls.

8. It would have been interesting to include a known substrate of NEXT in the analysis presented in Fig. 3A.

We appreciate this comment. However, we believe that the focus of the manuscript is not the NEXT complex and any additional result will confuse the reader. We use the **rrp4-2** ***and*** **cer7-3** ***mutants in the manuscript to convey the message that*** **hen2** ***mutants act independently of the core nuclear exosome. Further investigation of how the NEXT complex is involved in the cold response is an obvious future project.***

9. Fig 2, S1. It would be interesting to understand whether the cold-sensitivity is indeed linked to the decrease in 3' transcription of CBFs (or perhaps the upregulation of 5' regions). Do exosome mutants or ZCHCC8A/B share the cold-sensitive phenotype?

This is an interesting finding, but we believe this issue is outside the scope of the paper. The complexity of the involvement of micro RNAs and their dependency of the NEXT complex in the cold response makes any conclusions challenging to draw without a full investigation.

26-Aug-2024
Dr. Peter Kindgren
Umea Plant Science Centre
Forest Genetics and Plant Physiology
Umeå
Sweden

MSID: PP2024-RA-00978DR1
MS TITLE: The nuclear exosome subunit, HEN2, acts independently from the core exosome to assist transcription in Arabidopsis.

Dear Dr. Peter Kindgren:

I hope that you are well.

I am pleased to inform you that your revised manuscript "The nuclear exosome subunit, HEN2, acts independently from the core exosome to assist transcription in Arabidopsis." has been accepted for publication in Plant Physiology. Your manuscript will be checked for consistency with journal formatting and image standards by one of our Science Editors, who will contact you shortly. They may have a number of queries and suggestions for you. Please keep in mind that they work to increase the online visibility of your article and its attraction for readers from the widest of backgrounds. You will have the opportunity to review any revisions, and I hope you will work constructively with them to get the most out of your research. You will also have the opportunity to review all changes when you receive the author proofs for your article.

Once validated for publication, your article will be sent to a Science Editor for a final review of your manuscript and source files. After your final files are uploaded, your article will appear in the next available issue and the online Preview version of your article will be posted after your signed Copyright and Checklist forms and final source files are received.

Please be sure to include the following in your manuscript file:
Harvard style citations
List of all author contributions and funding information at the beginning of manuscript after the titles and author list.
List all supplemental materials associated with the article, with titles, at the end of the ms, before the references and acknowledgments section.
Complete Figure Legends for all figures appearing in the article listed before the Literature Cited.

LICENSE INFORMATION Shortly after your paper is sent to production, you will receive an email from our publisher's author support team, **SciPris**, with information on article processing charges, as well as the types of Open Access license you would be able to purchase. The accepted version of your manuscript will not appear on our Advance Articles page until the license is selected and signed, so please look out for an email from SciPris. For more information see the Article Publication Fees section of our author guidelines: https://academic.oup.com/plcell/pages/General-Instructions

Finally, we encourage your submission of artwork for the journal cover. If there is an image or illustration related to your paper that you would like to have considered, please email it in .pdf or .tif format to clowe@aspb.org. An image title, brief, 2-3 sentence description of the image, and the name of the person credited for the image are also encouraged. If your image is selected to appear on the online cover of the journal, you will be contacted with the scheduled issue date and further information.

Thank you and congratulations on your Plant Physiology paper!

Sincerely,
Editor, Plant Physiology

=====
FIRST AUTHOR PROFILES
=====

We recognize the hard work that goes into being First Author of a Plant Physiology paper by publishing a "First Author Feature" for each First Author alongside your paper.

If the first author(s) would like to submit information for a feature on Facebook and Twitter, fill out the form by clicking on the link below:

Plant Physiology First Author Feature Form: https://forms.office.com/r/ZBCGZfhm8F
If you cannot access the form through the link above, please contact Rachel at rbelsky@aspb.org.

To upload an image of yourself to accompany your profile, please put your photo in the correct journal folder at the link below. You must label your image: Author First Name Last Name Article Title.

First Author Profile Image Dropbox: https://www.dropbox.com/scl/fo/xz029p7qjr2jml2rp2kmq/h?dl=0&rlkey=6fkxcaqmxx35ap152b2osy1fz

=====
IMPORTANT REMINDER: PEER REVIEW REPORTS
=====

If you opted to publish a peer review report along with your article during the original submission process, it will be prepared by the editorial staff and publicly posted with your manuscript, inside the zip file that contains any other supplemental material. As a reminder, the peer review report is a public record of all comments from editors and reviewers, as well as your prior responses, as you received them in the decision letters for each draft of your manuscript. If you agreed to publish this report and have changed your mind, or are not sure if you selected this option, please contact the editorial office as soon as possible before signing the license agreement from our publisher.

-- Reviewer comments:
